# Antioxidant Effects of Hemp (*Cannabis sativa* L.) Inflorescence Extract in Stripped Linseed Oil

**DOI:** 10.3390/antiox9111131

**Published:** 2020-11-14

**Authors:** Carolina Cantele, Marta Bertolino, Fatema Bakro, Manuela Giordano, Małgorzata Jędryczka, Vladimiro Cardenia

**Affiliations:** 1 Department of Agricultural, Forest and Food Sciences (DISAFA), University of Turin, Grugliasco (TO) 10095, Italy; carolina.cantele@unito.it (C.C.); marta.bertolino@unito.it (M.B.); manuela.giordano@unito.it (M.G.); 2 Institute of Plant Genetics, Polish Academy of Sciences, Strzeszynska Str. 34, 60-479 Poznan, Poland; fbak@igr.poznan.pl (F.B.); mjed@igr.poznan.pl (M.J.)

**Keywords:** *Cannabis*, terpenoids, antioxidants, lipid oxidation, linseed oil

## Abstract

The ability of hemp (*Cannabis sativa* L.) inflorescence extract to counteract lipid oxidation was studied in stripped linseed oil. The ethanolic extract was characterized in terms of terpenes (6.00 mg/mL), cannabidiol (4.99% *w/w*), phenolic compounds (1.80 mg gallic acid equivalents (GAE)/mL), antiradical, and metal ion-chelating activities (50% effective concentration (EC_50_) of 2.47 mg/mL and 0.39 mg/mL, respectively). The stripped linseed oil, used as control (CO), was mixed with hemp extract (HO) or α-tocopherol (EO) at a ratio of 0.6% (*w/w*) and stored for 7 days in darkness at 40 °C. Hemp extract reduced the oxidation and lipolysis processes. At the end of the storage, HO showed a significantly higher level of α-linolenic acid (ALA; 26.64 g/100 g), lower peroxide value (PV) (21.19 meq O_2_/kg oil), and lower hexanal content (7.67 mmol/kg oil) than those found in the control. In contrast, EO showed a marked lipolysis (the free fatty acids increased by 42.57%) and a noticeable oxidation, since the ALA content decreased by 2.10% and a PV of 50 meq O_2_/kg oil was observed. This study demonstrates that hemp inflorescences can be used as a source of natural antioxidants in vegetable oils and lipid products to retard their oxidation, especially those characterized by a high degree of unsaturation.

## 1. Introduction

The consumption of polyunsaturated fatty acids (PUFAs) is generally promoted, since they play a key role in human health. In particular, it has been found that a higher intake of PUFAs prevents different chronic inflammatory processes and diseases, such as cancers, hypertension, cardiovascular diseases (CVDs), rheumatoid arthritis, and neurodegenerative processes [1,2]. The fatty acids are crucial components of cell membrane phospholipids; their composition affects the intercellular communication and autocrine signaling, also impacting gene expression [3]. A ratio higher than 4 of n-6 to n-3 PUFAs is linked to inflammation and CVDs, while a lower ratio is associated with reduced risk of chronic and inflammatory processes [4]. In general, the latest nutritional recommendations suggest increasing daily PUFA consumption, suggesting an uptake of linoleic acid (LA) and α-linolenic acid (ALA) at about 4.0% and 0.5% of daily energy intake, respectively [5].

The main sources of PUFAs are vegetable oils, such as linseed, hemp seed, almond, soybean, and sunflower oil, in addition to algae and fish oils [6]. Vegetable oils are finding more and more commercial applications; however, their susceptibility to lipid oxidation is a major concern for their use [7]. This impacts the quality oils and fats, reducing their nutritional value, since the oxidation products exert negative effects on human health, as well as impacting their sensory properties [8]. Many factors impact lipid oxidation, such as fatty acid composition and their degree of unsaturation [9], presence of pro-oxidant or antioxidant species, their solubility/lipophilicity, and storage conditions [10,11]. In order to counteract lipid oxidation in edible oils, different strategies have been employed. In particular, use of antioxidants capable of neutralizing free radicals and/or chelating metal ions represents an efficient strategy to extend the shelf life of vegetable oils, especially those characterized by a high degree of unsaturation. Butylated hydroxyanisole (BHA), *tert*-butylhydroquinone (TBHQ), and butylated hydroxytoluene (BHT) are the predominant synthetic antioxidants. However, since they can promote DNA damage [12,13] and due to health concerns from consumers, the use of natural antioxidants is preferred [8,14]. In addition, natural antioxidants may positively affect human health (e.g., cardioprotective effect, anticancer, etc.) giving added value to the edible oils [15]. Recently, greater interest has been addressed to hemp (*Cannabis sativa* L.), also called industrial cannabis (content of psychoactive cannabinoids <0.2%), mainly investigated due to the presence of more than 133 cannabinoids and terpenes [16,17]. However, hemp produces other metabolites, which could demonstrate antioxidant activity. Pollastro et al. [18] reported that hemp synthesizes characteristic molecules, such as prenylated flavonoids, stilbenoid derivatives, and lignamides, which require further investigations. Other studies [19,20] reported high amounts of polyphenols in hempseed cultivars Finola, Futura, Felina, and Carmagnola (Italy) with antioxidant activity. A special role is attributed to flavonoids, acting as free-radical scavengers and chelators of metal ions. Thus, the seeds of hemp are widely employed in the food industry, while the remaining inflorescences, considered as by-products, are mainly used for obtaining essential oils [21,22]. The cultivar Carmagnola was regarded as the most promising, due to the highest amount of polyphenols (about 743.5 mg/kg) in its inflorescences. They are an attractive source of natural antioxidants for nutraceutical purposes [23]. However, the hemp extracts were mainly chemically characterized and tested in vitro, while few data are available on their antioxidant role in bulk oil [16]. Therefore, the effect of the whole phyto-complex obtained from hemp inflorescences on lipid oxidation requires further deep investigation. 

The aim of the present work is to evaluate the impact of a hemp inflorescence extract on lipid oxidation of stripped linseed oil, which can provide fundamental knowledge to be implemented for improving the oxidative stability of high-PUFA edible oils. 

## 2. Materials and Methods 

### 2.1. Chemicals

Folin–Ciocalteu reagent, Cannabis Terpenes Mix A (2000 μg/mL in methanol of α-bisabolol, camphene, camphor, 3-carene, α-cedrene, cedrol, citronellol, fenchol, fenchone, geranyl acetate, α-humulene, isoborneol, limonene, menthol, cis-nerolidol, α-pinene, β-pinene, pulegone, α-terpinene, γ-terpinene), Cannabis Terpenes Mix B (2000 μg/mL in methanol of borneol, camphor, 3-carene, β-caryophyllene, *p*-cymene, β-eudesmol, geraniol, limonene, linalool, cis-nerolidol, β-pinene, phytol, α-terpinol, terpinolene), cannabidiol (CBD, 1 mg/mL in methanol), 5α-cholestane (≥ 97%), (±)-α-tocopherol (≥ 96%), γ-tocopherol (≥ 96%), linoleic acid conjugated methyl esters (cis-9,trans-11, trans-9,cis-11, trans-10,cis-12, cis-10,cis-12), methyl nonadecanoate (≥98%, C19:0), tridecane (≥99%, C_13_), 2-2′-diphenyl-1-picrylhydrazyl (DPPH^∙^), 6-hydroxy-2,5,7,8-tetramethylchroman-2-carboxylic acid (97%) (Trolox), ferrozine, silicic acid (100–200 mesh, 75–150 µm, acid washed), activated charcoal, chloroform (≥99%), and absolute ethanol were obtained from Sigma-Aldrich (Milan, Italy). The standard mixture of fatty acid methyl esters (GLC 463) was supplied by Nu-Chek (Elysian, MN, USA). Barium chloride (>99%), ethylenediaminetetraacetic acid tetrasodium salt dihydrate (EDTA), and gallic acid (≥98%) were provided by Fluka (Milan, Italy). Iron (II) sulfate heptahydrate was from Merck (Darmstadt, Germany). Sodium carbonate (Na_2_CO_3_), *n*-hexane, and potassium hydroxide (KOH) were purchased from Carlo Erba (Milan, Italy). The peroxide reagent kit was supplied by CDR S.r.l. (Florence, Italy). 

### 2.2. Preparation of the Ethanolic Extract 

Dried hemp inflorescences (European Union (EU) registered *C. sativa* L. Carmagnola variety; fiber-type), provided by a local company (Società Agricola FACT s.s., Moncalieri, Italy), were ground, and 1 g was extracted with 10 mL of ethanol absolute at room temperature as reported in Bakro et al. [24]. Briefly, the samples were stirred for 15 min (350 oscillations/min) and then sonicated for 20 min; after centrifugation (10 min; 8000× *g*), the supernatant was filtered through a Millipore filter (Polytetrafluoroethylene, PTFE; 0.45 µm). For simultaneous determination of terpenes and cannabidiol, 66 µL of a stock C_13_ standard solution (1000 µg/mL; internal standard) was added to the ground inflorescences before extraction with solvent.

### 2.3. Characterization of Hemp Inflorescence Extract

#### 2.3.1. Terpene Profile and Cannabidiol (CBD) Content

The terpene profile, as well as CBD content present in extract, was determined by fast gas chromatography with flame ionization detection (GC–FID) (Shimadzu QP 2010 Plus, Kyoto, Japan), according to our previous validated method [24]. The compounds were completely separated using helium as the carrier gas (linear velocity of 46.0 cm/s) on a fused capillary column (Restek RTX-5, 10 m × 0.1 mm inner diameter (id) × 0.1 µm film thickness) and with an oven temperature program of 60 °C (held for 1.30 min) ramping to 220 °C at a rate of 11.0 °C/min. The injector and detector temperatures were set at 310 °C and 340 °C, respectively. The identification of terpenes and CBD was established by comparing their retention times with those of commercial standards; their quantification by calibration curves built according to an internal standard method was achieved. The analysis was performed in triplicates.

#### 2.3.2. Total Phenolic Content (TPC)

TPC was determined by the Folin–Ciocalteu colorimetric method [25], adapted for a BioTek Synergy HT spectrophotometric multi-detection 96-well microplate reader (BioTek Instruments, Milan, Italy). Briefly, 20 μL of sample was mixed with 100 μL of Folin–Ciocalteu aqueous reagent (10%; *v/v*) and, after 3 min of incubation in the dark at 25 °C, with 7.5% (*w/v*) Na_2_CO_3_ solution. The mixed solution was incubated again in the dark at 25 °C for 1 h, and the absorbance was measured at 740 nm against blank controls. A standard curve of gallic acid (100–600 μM) was used to perform the quantification, and results were expressed in terms of milligrams of gallic acid equivalents (GAE) per milliliter of extract.

#### 2.3.3. Radical-Scavenging Activity (RSA)

The RSA of the hemp extract was spectrophotometrically assessed according to the DPPH^∙^ assay using a BioTek Synergy HT 96-well microplate reader [26]. The inhibition capacity of the sample was calculated and compared to a negative control in which the sample was replaced by ethanol [26]. The RSA was expressed as the concentration of the extract able to inhibit 50% of the initial DPPH^∙^ concentration (EC_50_), obtained by plotting the inhibition percentage of five different concentrations of the extract (1.82–9.10 mg/mL). Trolox was used as positive control (12.5–300 μM).

#### 2.3.4. Metal Ion-Chelating Activity

The metal-chelating ability of the hemp extract was determined with the ferrous ion-chelating assay [27]. The principle is based on the ability of ferrozine to chelate Fe^2+^ ions, forming a purple-colored complex which absorbs at 562 nm. One milliliter of extract (0.02–0.24 mg/mL) was mixed with 20 μL of FeCl_2_ (2 mM) and stirred vigorously; 20 μL of ferrozine (6 mM) was added and then stirred vigorously again. A negative control was also prepared, in which the sample was replaced by ethanol. After incubation (10 min), 200 μL of the solution was placed in a 96-well microplate reader, and the decrease in the absorbance was read at 562 nm compared against the negative control. Since Fe^2+^ it is easily oxidized, a fresh FeCl_2_ solution was daily prepared by reacting BaCl_2_ with FeSO_4_. The inhibition percentage was calculated according to the following equation:

% Inhibition = ((Abs*_Blk_* − Abs*_sample_*)/Abs*_Blk_*) × 100,

where Abs*_Blk_* is the absorbance of the negative control, and Abs*_sample_* is the absorbance of the sample. Metal-chelating activity was expressed as EC_50_ (the concentration capable of complexing 50% of the initial Fe^2+^ ion concentration), extrapolated by plotting the inhibition percentage of four different concentrations of the extract (0.02–0.24 mg/mL). EDTA was used as positive control (0.001–0.01 mg/mL).

### 2.4. Preparation of Stripped Linseed Oil

Cold-pressed linseed oil was supplied by a local company (F.lli Ruata Spa, Baldissero d’Alba, Italy) and stripped according to Boon et al. [28]. In brief, 30 g of linseed oil was dissolved in *n*-hexane (1:1; *w/v*) and passed through a chromatographic column (4.0 cm of diameter, 30 cm in length) packed with three layers: the top and bottom layers were silicic acid (22.5 g), while activated charcoal (5.625 g) was used in the middle layer. The column was then eluted with *n*-hexane (270 mL) in order to ensure the complete passage of the oil through the column. The solvent was evaporated under vacuum in a rotary evaporator (Rotavapor, R-210, Buchi, Flawil, Switzerland) at 37 °C. The effectiveness of the stripping procedure was ascertained by measuring the amount of tocopherols by reverse-phase high-performance liquid chromatography coupled to a photodiode array detector (RP-HPLC–PDA; Thermo-Finnigan, Waltham, MA, USA) [28]. The stripped linseed oil was used as the representative bulk oil in all experiments.

### 2.5. Bulk Oil Model System: Sample Preparation and Storage Conditions 

The α-tocopherol or hemp inflorescence ethanolic extract was placed into a beaker and, after evaporation of solvent with nitrogen gas, the stripped linseed oil was added at an extract/α-tocopherol:oil ratio of 0.6% (*w/w*). The stripped linseed oil containing hemp extract (HO), α-tocopherol (EO), or control oil without addition (CO) was then divided into 1 mL aliquots and placed into acid-washed 20 mL headspace vials, which were immediately sealed with aluminum caps with PTFE/silicone septa. The vials were stored in the dark at 40 °C for a week and analyzed after 0.25 (6 h), 1, 2, 3, 5, and 7 days of storage. The ratio and temperature were selected on the basis of our preliminary studies and the literature [10,29]. The obtained results were compared with those of fresh bulk oils. Three independent experiments (*n* = 3) were conducted.

### 2.6. Evaluation of Oxidative Stability of Bulk Oil 

#### 2.6.1. Determination of Main Lipid Classes 

The lipid profile of bulk oils as related to storage was determined according to our previous published work [30]. Twenty milligrams of oil were dissolved in 1.000 mL of chloroform containing 1.123 mg of cholestane (internal standard; IS), and 1 µL was injected into a GC–FID Shimadzu GC-2010 (Kyoto, Japan). The injection was performed in split mode (1:50), using helium as a carrier gas with a linear velocity of 47 cm/s and a fused silica Rtx-5 capillary column (10 m × 0.1 mm, film thickness 0.1 μm; Restek, Bellafonte, PA, USA) for separation. The oven temperature was ramped from 100 to 350 °C (at 5 °C/min) and then held at 350 °C for 20 min. The injector and detector temperatures were 348 °C and 350 °C, respectively. Identification of the main lipid classes (free fatty acids (FFAs), monoacylglycerols (MAGs), diacylglycerols (DAGs), triacylglycerols (TAGs), and esterified and free sterols) was performed by injecting mixtures of commercial standards under the same analytical conditions. The quantification was carried out with the internal standard method, and results were expressed as g/100 g oil.

#### 2.6.2. Total Fatty Acid Composition 

Twenty milligrams of oil combined with 1 mg of methyl nonadecanoate (C19:0; IS) dissolved in 1 mL of *n*-hexane was transesterified with 40 µL of KOH (2N) dissolved in methanol [31]; 1 µL of supernatant was injected into a GC–FID Shimadzu GC-2010 (Kyoto, Japan). The temperatures of the injector and detector were 245 °C and 250 °C, respectively. The linear velocity of the helium (carrier gas) was 22.4 cm/s. A fused silica SP-2560 capillary column (100 m × 0.25 mm, film thickness 0.2 μm; Supelco, Bellafonte, PA, USA) was used; the oven temperature was increased from 60 °C to 248 °C (4 °C/min) and was then maintained for 20 min. The different fatty acid methyl esters (FAMEs) were identified by comparing their retention times with those obtained from injecting the GLC 463 standard mixture under the same conditions. The quantification was achieved by the internal standard method and with the response factor of each FAME. Results were expressed as g/100 g oil.

#### 2.6.3. Determination of Peroxide Value

The content of peroxides in oil was determined with the *FoodLab* method (CDR S.r.l., Florence, Italy) [32]. In brief, a small aliquot of the sample (2.5–50 μL, depending on the concentration of peroxides) was mixed with the two kit reagents in a cuvette, and, after 3 min of incubation in the dark, the absorbance (510 nm) was measured, with the result expressed as milliequivalent of O_2_ per kilogram of oil (meq O_2_/kg oil). The upper limit of detection was equal to 50 meq O_2_/kg oil. According to Wang et al. [33], the protection factor (Pf) of α-tocopherol and hemp extract was also calculated as follows:

Pf = IP _oil samples with antioxidants/_IP _oil sample without antioxidants_

where IP corresponds to the induction period, which is the time required to reach a PV equal to 20 meq O_2_/kg oil. The protection factor determines whether a compound has antioxidant (Pf > 1) or pro-oxidant (Pf < 1) potential.

#### 2.6.4. Determination of Total Volatile Compounds (VOCs) 

Volatile compounds were isolated in the bulk oils (CO, HO, and EO) by headspace solid-phase microextraction (HS-SPME) using an autosampler COMBI PAL System (CTC Analytics AG, Zwingen, Switzerland) and then determined through a GC/MS Shimadzu QP-2010 Plus (Kyoto, Japan) as reported by Barbosa-Pereira et al. [34]. For the extraction of VOCs, a fiber coated with divinylbenzene/carboxen/polydimethylsiloxane (DVB/CAR/PDMS) (d_f_ 50/30 μm; 1 cm; Supelco, Bellafonte, PA, USA) was used. In order to reach the equilibrium inside the vial, samples were conditioned at 60 °C with continuous stirring at 250 rpm for 10 min. Then, the fiber was exposed to the headspace for 30 min in continuous agitation and heating. Next, the fiber was immediately desorbed in splitless mode into the GC/MS injector at 250 °C for 1 min. Helium was used as the carrier gas with a linear velocity of 36.5 cm/s. A fused silica DB-WAX capillary column (30 m × 0.25 mm, film thickness 0.25 μm; Agilent Technologies Inc., Santa Clara, CA, USA) was used to perform the separation with the following oven temperature conditions: 65 °C held for 5 min, then raised (4 °C/min) to 240 °C and maintained for 10 min. The temperatures of the transfer line and ion source were 230 °C and 200 °C, respectively. The mass acquisition range was 33–350 *m/z* with a scan velocity of the detector equal to 1166 amu/s. Compounds were identified by comparing their mass spectra with those contained in the NIST08s (National Institute of Standards and Technology, Gaithersburg) library. In addition, in order to prevent environmental contamination, a blank injection of fiber and vials was also carried out. The VOC data were reported as average peak area except for hexanal. Since hexanal was used as secondary lipid oxidation marker, a calibration curve was generated using hexanal standard solution (external standard method), and data were reported as mmol/kg oil.

### 2.7. Statistical Analysis

All results were reported as means and standard deviation of three independent replicates (*n* = 3). One-way ANOVA with Duncan’s post hoc test at a 95% confidence level was used to analyze the results and determine statistical differences. Factorial analysis of variance (ANOVA) was carried out to investigate the impact of lipid oxidation on the main lipid classes, fatty acids, terpenes, CBD, PV, and hexanal, as well as their interactions. Principal component analysis (PCA) was also performed on all volatile compounds data in order to better understand the data variability. IBM SPSS statistical software (version 25; IBM, Chicago, IL, USA) and GraphPad Prism 8.0 (GraphPad Software Inc. San Diego, CA, USA) were used to process the data. 

## 3. Results

### 3.1. Characterization of Hemp Inflorescence Extract

#### 3.1.1. Total Phenolic Content (TPC), Terpene Profile, and Cannabidiol Content

**Figure 1:**
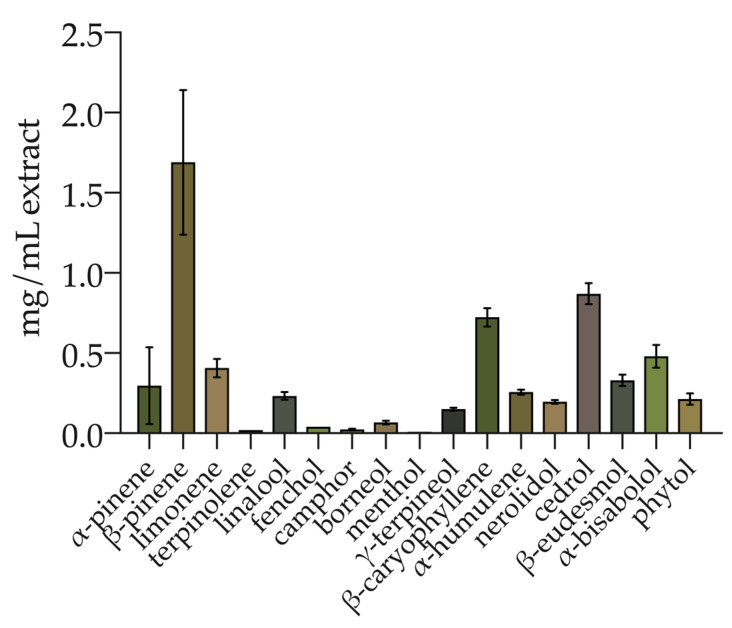
Terpene profile of hemp inflorescence ethanolic extract. Values are reported as the mean ± standard deviation (*n* = 3).

The total phenolic compounds were determined spectrophotometrically. The hemp ethanolic extract displayed a TPC of 1.80 ± 0.25 mg GAE/mL (equal to 19.73 ± 3.04 mg GAE/g inflorescence). In addition, the terpenes and CBD were simultaneously determined. Seventeen terpenes were identified and quantified (Figure 1) with a total amount of 6.00 ± 0.73 mg/mL of extract. The monoterpenes were 48.76% of total terpenes, while sesquiterpenes and diterpenes represented 47.65% and 3.59%, respectively. Among the monoterpenes, β-pinene, limonene, α-pinene, linalool, γ-terpineol, borneol, fenchol, camphor, terpinolene, and menthol were identified. Sesquiterpenes included cedrol, β-caryophyllene, α-bisabolol, eudesmol, α-humulene, and cis-nerolidol. Phytol was the only diterpene identified. The most abundant compound was β-pinene (1.69 ± 0.45 mg/mL; 28.17% of the total), followed by cedrol (0.87 ± 0.07 mg/mL; 14.5% of the total) and β-caryophyllene (0.72 ± 0.06 mg/mL; 12% of the total). The terpene profile is detailed in Table S1 (Supplementary Materials).

In addition, the content of cannabidiol (CBD; the main phytocannabinoid reported in hemp) was equal to 4.99% (*w/w*) ± 1.24%.

#### 3.1.2. Antioxidant Activity

The antioxidant properties of hemp inflorescence ethanolic extract were determined in terms of radical-scavenging activity (RSA) and chelating activity (CA) using Trolox and EDTA, respectively, as positive controls. With regard to the RSA, 2.47 ± 0.10 mg/mL of hemp extract was able to inhibit 50% of DPPH (EC_50_), which represented 35- fold the value detected in the positive control (0.07 ± 0.00 mg/mL). Furthermore, the hemp extract displayed a chelating activity, whereby 0.39 ± 0.01 mg/mL of extract was able to chelate 50% of ferrous ions (EC_50_), which was 35-fold higher than the positive control EC_50_ (0.01 ± 0.00 mg/mL).

### 3.2. Evaluation of the Oxidative Stability of Bulk Oil

#### 3.2.1. Main Lipid Classes

**Figure 2:**
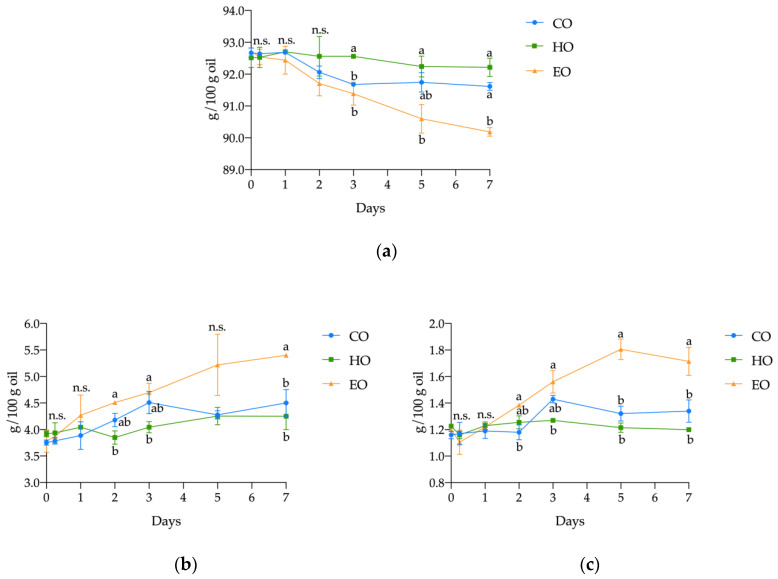
Kinetics of (**a**) triacylglycerols (TAGs), (**b**) diacylglycerols (DAGs), and (**c**) free fatty acids (FFAs) in stripped linseed oil without antioxidants (CO), with hemp extract (HO), and with α-tocopherol (EO) during storage. Values (g/100 g oil) are expressed as mean ± standard deviation (*n* = 3). Results of ANOVA with Duncan’s test are reported between CO, HO, and EO within the same day. Different letters a, b are significantly different at *p* < 0.05. n.s. = not significant

Regarding the main lipid classes, significant differences were found in the content of triacylglycerols (TAGs), diacylglycerols (DAGs), and free fatty acids (FFAs) in pure stripped linseed oil (CO), with added hemp extract (HO) or α-tocopherol (EO) during the 7 days of treatment (Figure 2). Both CO and HO displayed a similar hydrolytic behavior. In fact, during the whole treatment, both CO and HO displayed similar content of TAGs, DAGs, and FFAs. On the other hand, in EO, the content of TAGs significantly decreased from a starting content of 92.72 ± 0.07 g/100 g (*p* < 0.01; Table S2, Supplementary Materials) over the course of the experiment, finally reaching a value of 90.18 ± 0.14 g/100 g after 7 days, which corresponds to a reduction of 2.73%. Conversely, an opposite pattern was observed for DAGs and FFAs. In EO, the highest values of FFAs were found; from 1.20 ± 0.02 g/100 g at day 0, they increased to 1.71 ± 0.11 g/100 g at the end of the experiment, which was a significant increase (*p* < 0.001; Table S2, Supplementary Materials) of 42.57%. The DAGs also showed the most pronounced increase (42.66%) (*p* < 0.01; Table S2, Supplementary Materials), from 3.78 ± 0.21 to 5.40 ± 0.04 g/100 g.

In addition to TAGs, DAGs, and FFAs, the free and esterified sterols were also quantified (Table S2, Supplementary Materials); however, no significant differences (*p* > 0.05) were observed in bulk oil composition.

Alongside the lipid classes, the content of CBD was also monitored in HO since it might show an antioxidant effect. However, it remained unchanged until the last day (*p* > 0.05) (Table S3, Supplementary Materials).

#### 3.2.2. Total Fatty Acid Composition

In order to better define how the lipid oxidation impacted the bulk oils, the total fatty acid methyl ester (FAME) composition of samples was determined. Only the fatty acids representing more than 0.1% of the total FAMEs were considered, except for the sum of conjugated linoleic acid isomers (CLA) and conjugated linolenic acid isomers (CLnA). 

The CO was mainly composed of polyunsaturated fatty acids (PUFAs; 71.46 ± 0.03 g/100 g oil), followed by monounsaturated fatty acids (MUFAs; 17.82 ± 0.01 g/100 g oil) and saturated fatty acids (SFAs; 10.19 ± 0.03 g/100 g oil) (Table 1). Among the PUFAs, LA (C18:2 n-6) was the most abundant (44.62 ± 0.00 g/100 g oil of the total FAMEs), followed by ALA (C18:3 n-3; 26.70 ± 0.03 g/100 g oil). Oleic acid (C18:1) was the main MUFA (16.98 ± 0.00 g/100 g oil), while palmitic (C16:0) and stearic (C18:0) acids were the principal SFAs. Myristic acid (C14:0), pentadecylic acid (C15:0), palmitoleic acid (C16:1), margaric acid (C17:0), margaroleic acid (C17:1), the sum of trans-isomers, eicosadienoic acid (C20:2), eicosatrienoic acid (C20:3 n*-*3), arachidonic acid (AA; C20:4), heneicosylic acid (C21:0), erucic acid (C22:1), dihomo-γ-linolenic acid (DGLA; C22:6 n*-*6), docosahexaenoic acid (DHA; C22:6 n*-*3), lignoceric acid (C24:0), and nervonic acid (C24:1) were detected just in trace amounts, with their total accounting for 0.54 ± 0.01 g/100 g oil (Tables S4, S5, and S6, Supplementary Materials). 

On the basis of results, no significant changes in SFAs, MUFAs, and PUFAs were observed in HO (*p* > 0.05), while significant differences were observed for CO and EO. After three days, CO displayed a significant decrease in the PUFA content equal to 0.40%, while, in EO, the decrease was even more intense, with a significant reduction in PUFAs by 0.84% after 2 days. At the end of the experiment, the content of both saturated and unsaturated fatty acids was not different between CO and HO; however, there was a mild tendency of CO toward oxidation, displaying intermediate values between HO and EO, except for MUFAs.

**Figure 3:**
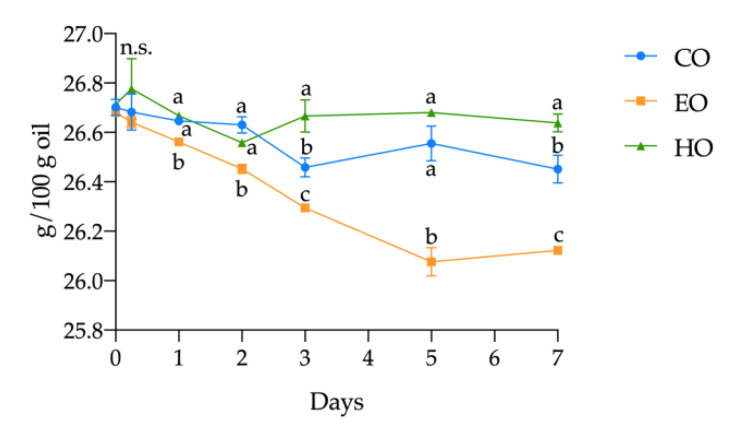
Kinetics of α-linolenic acid (ALA) in stripped linseed oil without antioxidants (CO), with hemp extract (HO), and with α-tocopherol (EO) during the storage treatment. For each point (mean ± standard deviation of three independent replicates), results of ANOVA with Duncan’s test are reported between the samples within the same day. Different letters a–c mean the results were significantly different at *p* < 0.05. n.s. = not significant.

Considering the single FAME, in HO, no significant changes (*p* > 0.05) were observed during the treatment. On the other hand, in EO an extensive reduction in ALA and LA content (by 2.10% and 0.19%, respectively) was observed. In CO, the decrease in ALA was quantified as 0.93%, while LA remained unvaried over time (*p* > 0.05). However, the oxidation in CO was less severe compared to EO, confirming once again the pro-oxidant effect of α-tocopherol found in this study. The progress in ALA oxidation is shown in Figure 3. Since the kinetics of ALA is an exact reflection of that of the PUFAs in all the three samples, and since the other PUFAs remained stable, it can be established that the decrease in PUFA content was mainly due to the degradation of ALA.

#### 3.2.3. Peroxide Value (PV)

**Figure 4:**
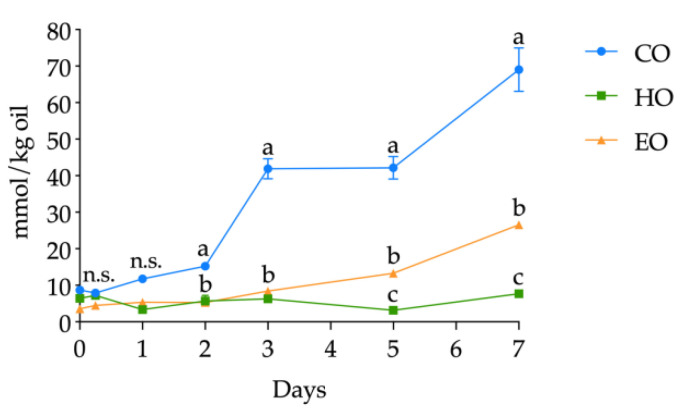
Kinetics of peroxide value (PV) (meq O_2_/kg oil) in stripped linseed oil without antioxidants (CO), with hemp extract (HO), and with α-tocopherol (EO) and results of ANOVA with Duncan’s test. Each point corresponds to the mean ± standard deviation (*n* = 3). Different letters a,b within the same day mean significantly different results at *p* < 0.05 among different samples. n.s. = not significant.

The kinetics of PV in CO, HO, and EO oil samples during the seven days of storage is depicted in Figure 4. At day 0, the three bulk oils displayed similar PV (*p* > 0.05) equal to 2.45 ± 0.02, 3.26 ± 0.18, and 3.91 ± 0.66 meq O_2_/kg oil for CO, HO, and EO, respectively (see Table S7, Supplementary Materials, for detailed PV). Afterward, EO displayed a very different pattern with respect to CO and HO. In fact, while CO and HO showed a lag phase of one day (*p* > 0.05 between day 0 and day 1) (Table S7, Supplementary Materials), in EO after 6 h of storage, the PV was twice the value found in CO and HO; again, after 1 day, the PV was threefold higher and reached the highest amount of 45.46 ± 0.74 meq O_2_/kg oil on day 2. It should be pointed out that PV was used to define the lag phase; thus, after 2 days of storage, EO was not analyzed. 

With regard to CO and HO, until day 3, the PV was not significantly different (*p* > 0.05). However, from day 3 onward, their kinetics differentiated. In fact, the PV in CO was significantly higher than that detected in HO (*p* < 0.05), and, at day 7, the CO already reached the value of 49.57 ± 0.22 meq O_2_/kg oil, while, in HO, a PV of 21.19 ± 1.44 meq O_2_/kg oil was found. Moreover, PV in HO from day 3 to 7 remained indistinguishable (*p* > 0.05) (Table S7, Supplementary Materials).

According to the induction period (the time required to reach a peroxide value equal to 20 meq O_2_/kg oil (CO, 2 days; HO, 7 days; EO, 1 day) [33]), the resulting protection factor (Pf) was equal to 3.5 and 0.5 for HO and EO, respectively, attesting the antioxidant potential of the hemp extract.

#### 3.2.4. Volatile Compounds 

**Figure 5:**
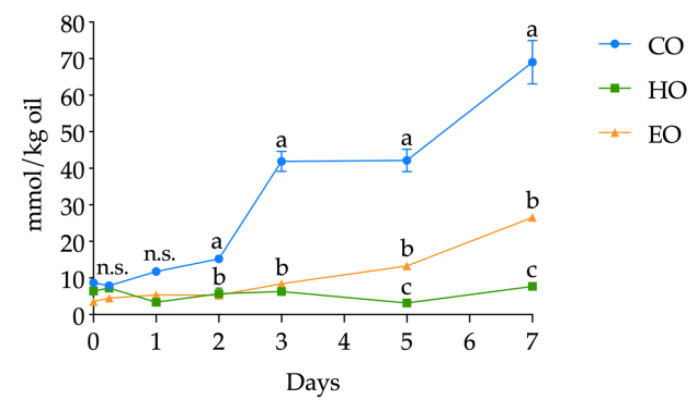
Kinetics of hexanal content (mmol/kg oil) in stripped linseed oil without antioxidants (CO), with hemp extract (HO), and with α-tocopherol (EO) and results of ANOVA with Duncan’s test. Each point represents the mean ± standard deviation of three independent replicates (*n* = 3). Different letters a–c within the same day are significantly different at *p* < 0.05. n.s. = not significant.

The volatile compounds were determined as related to bulk oils and storage time (detailed in Tables S9, S10, and S11, Supplementary Materials); however, on the basis of the literature, hexanal was selected as a lipid secondary oxidation marker and its quantification was carried out. The kinetics of hexanal levels in CO, HO, and EO is shown in Figure 5. The starting values of hexanal in fresh bulk oils ranged from 3.57 ± 0.60 to 8.71 ± 0.13 mmol/kg oil without significant differences up to day 1 (all the hexanal contents are shown in detail in Table S8, Supplementary Materials). At day 1, hexanal in CO was significantly higher (*p* < 0.01) than in HO and EO and, from this point forward, drastically increased during the whole storage treatment, displaying each day the highest value among the three bulk oils. Hexanal in EO increased after 3 days of storage (*p* < 0.01) (Table S8, Supplementary Materials) and reached its highest value of 26.53 ± 0.54 mmol/kg oil after 7 days. On the contrary, HO exhibited different kinetics from the two other oils. In fact, hexanal content in HO displayed no significant changes in abundance (*p* > 0.05) (Table S8, Supplementary Materials), keeping low values throughout the whole storage treatment.

In order to better define the impact of lipid oxidation on bulk oils, principal component analysis (PCA) of all volatile compounds during the whole experiment was carried out. PCA was able to explain 73.90% of the total variance in two principal components, highlighting the presence of different clusters. In the first principal component (PC1), which explains 59.60% of variance, the terpenes except for 3-carene, l-fenchone, and *p*-xylene were separated from 1-hexanol, 6 methyl-5-hepten-2-one, and butanoic acid. The second principal component (PC2; 13.80% of total variance) visibly separated hexanal and 2,4-nonadiene from nonanal, emphasizing their inverse correlation. A third cluster comprising formic acid, 2-butenal, α-pinene, and decane was observed, while β-pinene was disjointed from the remaining terpenoid compounds.

**Figure 6:**
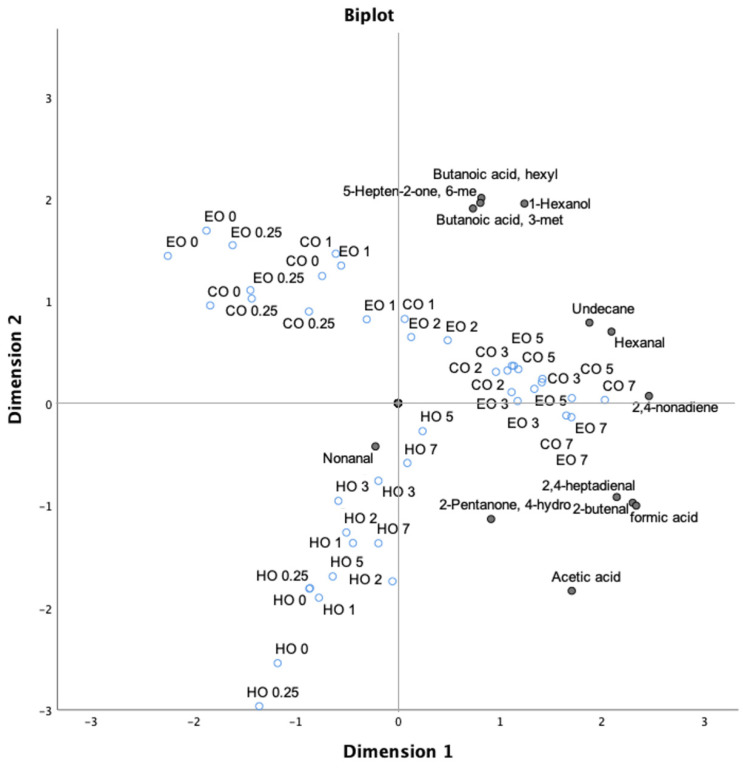
Bi-plot of volatile compounds (black circles), excluding the terpenoid compounds, and treatment time (empty blue circles) in thermo-oxidized bulk oils (stripped linseed oil without antioxidants (CO), with hemp extract (HO), and with α-tocopherol (EO)).

As reported in Figure 6, a clear oxidation trend was found for both CO and EO samples as related to time of storage, while the HO samples displayed a different behavior showing the impact of hemp extract in counteracting the lipid oxidation.

Again, when all volatile compounds were considered, including terpenoid compounds, two main clusters were exhibited. PC1 divided the HO samples from both CO and EO, which were mainly separated in relation to the time of storage by PC2. The clustering of HO samples was characterized by terpenes and, as mentioned above, no significant effects of VOCs originating from lipid oxidation were implicated, confirming the capacity of hemp extract to counteract the lipid oxidation in the tested bulk oil (Figure S1, Supplementary Materials). 

## 4. Discussion

The aim of this work was to evaluate the efficacy of hemp inflorescence ethanolic extract to protect a vegetable oil rich in polyunsaturated fatty acids (PUFAs), namely, linseed oil, against oxidation. Hemp is rich in bioactive compounds, particularly polyphenols and terpenes, potentially capable of counteracting reactive metals and free radicals and, therefore, we hypothesized, of retarding oxidation in vegetable oil. In this study, the bioactive compounds were extracted from inflorescences with ethanol, since it is generally established that this solvent is of less concern to human health than other organic solvents. This is why it is extensively used in the food sector and does not constitute an environmental issue [24]. Moreover, it is reported that ethanol offers the best performance in terms of extraction yields and antioxidant effects [35]. The hemp inflorescence ethanolic extract was investigated in order to profile the terpenoid compounds, estimate the total phenolic content (TPC), and determine its antioxidant activities. The amount of phytocannabinoids classified the hemp as “fiber-type” *Cannabis*, chemotype III, with a total content of Δ^9^-tetrahydrocannabinol (THC) <0.6% (which represents the legal limit in Italy [36]) and lower than that of CBD (ratio (THC + CBN)/CBD < 0.02 [37]), wherein CBN represents cannabinol content. 

The odor and flavor in *Cannabis* species are determined by terpenes, contributing to a characterization of the different strains [38]. In this study, the total amounts of monoterpenes and sesquiterpenes (96.41%) were in line with the literature, which reports up to 98% of these two families in cannabis essential oil [39]. The profile of the terpene classes was in agreement with that found by Nissen et al. [40] in the essential oil of the Carmagnola variety, while that of the single terpenes was different. In fact, the authors [40] reported β-myrcene (29.22%), α-pinene (15.12%), and β-caryophyllene (13.90%) as the main terpenes. In our study, the three most abundant were β-pinene, β-caryophyllene, and cedrol, whose health benefits (such as anti-inflammatory, antidepressant, and sedative activities, antihypertensive and relaxant effects, and pharmacological actions including cardioprotective, neuroprotective, gastroprotective, hepatoprotective, and immunomodulatory) have been widely described [38,39,41]. This discrepancy with Nissen et al. [40] is likely due to the difference in varieties, as well as growth factors such as soil and climate conditions, abiotic stress, harvesting time, and tissue type [24]. In addition to cannabinoids and terpenes, phenolic compounds are reported in hemp, including flavonoids (such as flavonols and flavones), lignamides (such as cannabisin-like compounds), and stilbenoids (such as cannabistilbene-like compounds) [18,38]. In particular, apigenin, chlorogenic acid, ferulic acid, luteolin, quercetin, *N*-trans-caffeoyltyramine, *p*-coumaric acid, cannflavin A, catechin, cannflavin B, epicatechin, and quercetin-3-glucoside (ranging 8.7–126.1 mg/kg) are the most recurrent polyphenols in the inflorescences of Carmagnola variety [23]. Polyphenols can play a fundamental role in human health, due to their antioxidant properties and their ability to modulate or inhibit certain enzymes such as lipoxygenases [42]. The TPC found in the present study was slightly lower compared with that reported by Izzo et al. [23] in the Carmagnola cultivar of hemp inflorescence extracted with methanol (ranged 26.3–41.5 mg GAE/g). However, it was higher than that documented by other researchers (Frassinetti et al. [43] with about 6 mg GAE/g in hemp sprouts, and Drinić et al. [35] with 6.43 and 5.85 mg GAE/g in 90% ethanol extracts of young and mature hemp inflorescences, respectively). Similarly to terpenes, the profile and content of flavonoids in cannabis are also influenced by factors other than the variety. In fact, their distribution varies enormously from plant to plant and according to the tissue considered, and, as the plant ages, their content decreases [18]. The method of extraction parameters (such as the type of solvent, the extraction time and temperature, and the particle size of the sample) are also influential in a solid–liquid extraction [35]. Indeed, Izzo et al. [23], despite having investigated the same variety as this study, obtained a higher TPC value, since they used methanol as a solvent, which is one of the most effective extractants, but much more toxic than ethanol. 

In comparison with the literature, it might be pointed out that the inflorescence extract is more able to scavenge DPPH radicals and chelate ferrous ions than hemp leaves and seeds. In fact, as reported by the authors [44], hemp leaves and seeds displayed a relatively higher EC_50_ for both RSA and chelating activity (a higher EC_50_ value indicates a lower activity) than that reported in the present study.

However, similar EC_50_ values were observed for the DPPH^∙^ assay in two different varieties of hempseed kernel extracted with 100% ethanol [45]. Nevertheless, the EC_50_ of DPPH^∙^ found in the present study was much higher than that reported by other research on the Carmagnola cultivar cultivated in different areas of the Po valley (Italy) [20]. However, as stated by Lesma et al. [20], the RSA is influenced not only by the variety, but also by the soil where it is grown and by the weather conditions, potentially explaining the discrepancy between RSA values even within the same cultivar.

Having established its potential as an antioxidant agent, the hemp inflorescence extract was then tested in a bulk oil model system, wherein stripped linseed oil was exposed to high temperatures in order to induce its rapid degradation. This process involves a series of chemical and enzymatic reactions that lead to triacylglycerol hydrolysis and fatty acid primary and secondary oxidation, causing a deterioration in the sensory and nutritional properties of the oil due to the formation of undesirable and toxic compounds [9,46,47].

On the basis of the composition of the main lipid classes, the hemp inflorescence extract cannot be regarded as an agent causing great resistance of the oil against lipolysis; no differences between HO and CO were noted until the last day. During the experiment, the hydrolysis in CO was not severe; a minimal cleavage of TAGs and DAGs (and consequent release of fatty acids) was observed, whereas, in HO, there was no such reaction. Usually, the hydrolysis of TAGs is carried out by enzymes, principally lipases and phospholipases. The latter are water-soluble and act at the interfaces with water naturally present in the oil and the lipids [48]. Lipolytic breakdown is determined mostly by the chemical–physical conditions of the oil, which are governed by upstream factors, such as the condition of the plant from which the oil has been extracted (which affects the concentration of lipase enzymes) and the conditions of the oil processing (which affects the moisture content) [48]. Since the stripping process inhibits lipase activity, the lipolysis that occurred in our samples can be mainly ascribed to chemical reactions. 

On the other hand, the inflorescence extract turned out to be an effective antioxidant, preventing both primary and secondary oxidation, with HO showing an increased oxidative stability compared to CO and EO. The protective effect of hemp was already evident from the composition of fatty acids, since all the unsaturated fatty acids exhibited a constant trend throughout the entire experiment, while, in CO and EO, a pronounced oxidation of the PUFAs occurred. In addition, the content of ALA in HO on day 7 was higher than in CO, demonstrating that the hemp extract reduced ALA oxidation. Unlike ALA, LA remained stable in both HO and CO. The most probable reason is that linolenate, having a higher degree of unsaturation, is more prone to oxidation than linoleate with 2.4-fold higher reactivity. Thus, linolenate is the first substrate hit by free radicals [9].

In contrast, EO exhibited completely different kinetics compared to CO and HO, starting from lipolysis. A strong hydrolysis of TAGs was followed by an increase in DAG and FFA content. Since the free PUFAs are much more prone to oxidation than their esterified counterparts [48], a severe worsening of the fatty acid composition took place in EO. Almost all FAMEs were affected by oxidation, with a drastic and significant reduction in ALA and even LA, underlining the more marked progress in oxidation. This pro-oxidant activity has been already described by the other researchers [33,49,50]. In fact, it was stated that many factors control the antioxidant or pro-oxidant effect of α-tocopherol, namely, the nature of the substrate and its mobility within it, its concentration, and the applied conditions, such as the temperature and the potential interactions with other substances in the matrix [51]. Moreover, it has been suggested that, in oils, the most effective antioxidants are polar antioxidants and not nonpolar ones, which are more effective in protecting emulsions (the so-called “polar paradox”) [52].

As expected, the protective effects of hemp inflorescences against oxidation, as well as the pro-oxidant effect of α-tocopherol, were established by primary and secondary oxidation products and the protection factor (Pf). According to Frankel [46], peroxide degradation takes place at very high temperatures, and this could be an explanation for the observed linear trend, since a moderate heating was applied in the experiment (40 °C). CO and HO were characterized, as expected, by a progress in hexanal formation that reflected the peroxide content. Thus, an increasing (CO) or constant (HO) trend in peroxide development led to an increasing (CO) or constant (HO) trend in hexanal formation. The trend of hexanal is also a reflection of ALA oxidation, since it is widely reported as a precursor [53]. Consequently, the progressive oxidation of ALA in CO resulted in a progressive increase in hexanal, while the stability of ALA in HO resulted in an unchanged hexanal content. On the other hand, a completely different process was observed in EO. In fact, despite reaching rancidity after only 2 days (50 meq O_2_/kg oil as the maximum value indicates that complete rancidity has been reached), the hexanal content remained unusually low. Other authors documented the same situation, stating that a higher concentration of α-tocopherol led to a higher inhibition of hexanal formation [53,54]. The authors suggested that this suppression is due to the action of α-tocopherol on both peroxyl and alkoxyl radicals. On one hand, α-tocopherol, competing with the lipid substrate, might donate its hydrogen atom to the alkoxyl radical, forming a more stable tocopheroxyl radical, which is then also able to trap the alkoxyl radical [54]. On the other hand, α-tocopherol could act on peroxyl radicals by intercepting them and reducing the decomposition of peroxides and, consequently, the formation of hexanal [53].

The conjugated fatty acids were examined through GC–FID and not by measuring the absorbance because, as reported by Tura et al. [16], the cannabinoids can interfere, leading to an overestimation of data. However, the sum of conjugated linoleic acids (CLAs) and conjugated linolenic acids (CLnAs) did not play an important role in the study, as their concentration was scarce and remained unchanged over time in all samples.

The consistency of all oxidation parameters in HO during storage could be due to the ability of hemp bioactive compounds to withdraw the metal ions and free radicals and, therefore, to prevent and delay, respectively, the free-radical chain reaction on fatty acids. These two abilities are governed predominantly by the phenolic compounds thanks to their molecular structure, which is the factor that most influences the RSA and the metal-chelating capacity [49]. The presence of a phenolic pattern and alkyl and hydroxyl groups on the phenol ring, together with a low bond dissociation energy of the latter (as occurs in apigenin, quercetin, luteolin, catechin, epicatechin, and cannflavin A and B), makes an antioxidant more efficient [49]. In addition, the efficacy of chelating metal ions is enhanced if certain functional moieties (i.e., two hydroxy groups, one hydroxy and one carboxyl group, or one hydroxy and one carbonyl group) are positioned on the carbon atoms adjacent to the phenol ring [49]. Furthermore, antioxidant activity was also demonstrated in terpenes, depending again on their molecular structure. In fact, Wojtunik et al. [55] demonstrated that DPPH^∙^ inhibition increased in the presence of π-conjugated bonds, which enabled formation of resonance-stabilized structures when reacting with the radical, delocalizing the charge over the whole molecule. None of the terpenes identified in this study presented conjugated double bonds; however, for some of them, such as linalool, limonene, terpinolene, and β-caryophyllene, a strong antioxidant activity was reported by other authors [56,57,58]. In addition, since the CBD did not degrade during the experiment in bulk oils and, as reported by Tura et al. [16], CBD does not protect fatty acids from oxidation, we can exclude the possibility that CBD is directly involved in the aforementioned antioxidant mechanisms. However, there is a possibility that CBD boosted the antioxidant activity of terpenes and/or polyphenols through a synergistic effect, since an enhancement of the antioxidant activity in hemp extracts was reported when cannabinoids were accompanied by a high amount of polyphenols [59].

## 5. Conclusions

Preserving polyunsaturated fatty acids (PUFAs), especially those with more than two double bonds such as α-linolenic acid (ALA), represents the key to good maintenance of vegetable oils and lipid-containing foods in general, since, from their oxidation, toxic compounds are produced, also affecting their flavor acceptability. In the present study, the ability of a hemp inflorescence extract to enhance the oxidative stability of a PUFA-rich vegetable oil, namely, linseed oil, was demonstrated. Although it had no impact on lipolysis, the hemp inflorescence extract was an efficient antioxidant, able to preserve the very unstable ALA and prevent the formation of peroxides and, consequently, hexanal. This protective effect against oxidation can be attributed to the bioactive compounds present in the inflorescences, mainly terpenes and polyphenols, which reduce free radicals and caused the chelation of metals, the main causes of the autocatalytic process of lipid oxidation. Further studies are required to better define the antioxidant mechanism of hemp extract, as well as the possible synergistic effect of terpenes, phenols, and cannabinoids.

## Figures and Tables

**Table 1:** Kinetics of total (g/100 g oil) saturated fatty acids (∑ SFAs), monounsaturated fatty acids (∑ MUFAs), and polyunsaturated fatty acids (∑ PUFAs) in CO, HO, and EO during 7 days of treatment.

		Day 0	Day 0.25	Day 1	Day 2	Day3	Day 5	Day 7	*Sig.*
∑ SFAs	CO	10.19 ± 0.03	10.21 ± 0.05	10.20 ± 0.03	10.22 ± 0.03	10.51 ± 0.10	10.28 ± 0.04 ^Y^	10.30 ± 0.05 ^XY^	n.s.
	HO	10.17 ± 0.02	10.15 ± 0.08	10.21 ± 0.04	10.33 ± 0.16	10.15 ± 0.22	10.22 ± 0.02 ^Y^	10.23 ± 0.08 ^Y^	n.s.
	EO	10.16 ± 0.00 ^d^	10.19 ± 0.02 ^d^	10.23 ± 0.03 ^cd^	10.28 ± 0.02 ^bc^	10.32 ± 0.04 ^b^	10.41 ± 0.05 ^aX^	10.42 ± 0.04 ^aX^	**
	*Sig.*	n.s.	n.s.	n.s.	n.s.	n.s.	*	*	
∑ MUFAs	CO	17.82 ± 0.01 ^b^	17.81 ± 0.07 ^b^	17.85 ± 0.01 ^bY^	17.86 ± 0.04 ^bXY^	17.87 ± 0.01 ^abY^	17.90 ± 0.04 ^abY^	17.96 ± 0.05 ^aY^	*
	HO	17.80 ± 0.00	17.77 ± 0.06	17.83 ± 0.01 ^Y^	17.80 ± 0.04 ^Y^	17.86 ± 0.01 ^Y^	17.82 ± 0.01 ^Y^	17.85 ± 0.04 ^Y^	n.s.
	EO	17.83 ± 0.00 ^d^	17.85 ± 0.00 ^d^	17.89 ± 0.01 ^cdX^	17.96 ± 0.02 ^cX^	18.05 ± 0.02 ^bX^	18.18 ± 0.06 ^aX^	18.16 ± 0.04 ^aX^	***
	*Sig.*	n.s.	n.s.	*	*	**	**	*	
∑ PUFAs	CO	71.46 ± 0.03 ^a^	71.41 ± 0.10 ^a^	71.41 ± 0.03 ^a^	71.38 ± 0.08 ^ab^	71.05 ± 0.13 ^c^	71.27 ± 0.10 ^abX^	71.17 ± 0.11 ^bc XY^	**
	HO	71.47 ± 0.03	71.52 ± 0.15	71.41 ± 0.06	71.29 ± 0.19	71.43 ± 0.21	71.40 ± 0.04 ^X^	71.37 ± 0.13 ^X^	n.s.
	EO	71.46 ± 0.00 ^a^	71.41 ± 0.03 ^a^	71.32 ± 0.03 ^ab^	71.21 ± 0.05 ^bc^	71.05 ± 0.07 ^c^	70.83 ± 0.12 ^dY^	70.86 ± 0.08 ^dY^	***
	*Sig.*	n.s.	n.s.	n.s.	n.s.	n.s.	*	*	

Results are reported as mean ± standard deviation (*n* = 3). Means followed by the same lowercase (row; within the sample, among the days) letters a–d and uppercase (column; within the day, among the samples) letters X,Y are not significantly different at *p* < 0.05 (Duncan’s test). n.s. = not significant; * = *p* < 0.05; ** = *p* < 0.01; *** = *p* < 0.001.

## Supplementary Materials

The following are available online at https://www.mdpi.com/2076-3921/9/11/1131/s1: Table S1. Terpenes profile of hemp inflorescences ethanolic extract; Table S2. Main lipid classes (g/100 g oil) in bulk oil without antioxidants (CO), with hemp extract (HO), and with α-tocopherol (EO); Table S3. Content of cannabidiol (CBD) (g/100 g oil) in bulk oil with hemp extract (HO); Table S4. Total fatty acid methyl esters (FAMEs; g/100 g oil) in bulk oil without antioxidants (CO), during the storage treatment; Table S5. Total fatty acid methyl esters (FAMEs; g/100 g oil) in bulk oil with hemp extract antioxidants (HO), during storage; Table S6. Total fatty acid methyl esters (FAMEs; g/100 g oil) in bulk oil with α-tocopherol (EO) during storage; Table S7. Peroxide value (meq O_2_/kg oil) in bulk oil without antioxidants (CO), with hemp extract (HO), and with α-tocopherol (EO); Table S8. Formation of hexanal (mmol/kg oil) in bulk oil without antioxidants (CO), with hemp extract (HO), and with α-tocopherol (EO); Table S9. Identified volatile compounds (as peak area amount) in thermoxidized stripped oil samples without hemp extract or α-tocopherol (CO) for 0, 0.25, 1, 2, 3, 5, and 7 days; Table S10. Identified volatile compounds (as peak area amount) in thermoxidized stripped oil samples containing hemp extract (HO) for 0, 0.25, 1, 2, 3, 5, and 7 days; Table S11. Identified volatile compounds (as peak area amount) in thermoxidized stripped oil samples containing α-tocopherol (EO) for 0, 0.25, 1, 2, 3, 5, and 7 days; Figure S1. Bi-plot of all volatile compounds (black squares) and treatment time (empty squares) in thermo-oxidized bulk oils.

## References

[1] Sioen I., van Lieshout L., Eilander A., Fleith M., Lohner S., Szommer A., Petisca C., Eussen S., Forsyth S., Calder P.C. (2017). Systematic review on N-3 and N-6 polyunsaturated fatty acid intake in European countries in light of the current recommendations—Focus on specific population groups. Ann. Nutr. Metab..

[2] Malaguti M., Cardenia V., Rodriguez-Estrada M.T., Hrelia S. (2019). Nutraceuticals and physical activity: Their role on oxysterols-mediated neurodegeneration. J. Steroid Biochem. Mol. Biol..

[3] SanGiovanni J.P., Chew E.Y. (2005). The role of omega-3 long-chain polyunsaturated fatty acids in health and disease of the retina. Prog. Retin. Eye Res..

[4] Simopoulos A.P. (2008). The importance of the omega-6/omega-3 fatty acid ratio in cardiovascular disease and other chronic diseases. Exp. Biol. Med..

[5] EFSA Panel on Dietetic Products Nutrition and Allergies (NDA) (2013). Scientific opinion on nutrient requirements and dietary intakes of infants and young children in the European Union. EFSA Eur. Food Saf. Auth. J..

[6] Harwood J.L. (2019). Algae: Critical sources of very long-chain polyunsaturated fatty acids. Biomolecules.

[7] Waraho T., Cardenia V., Rodriguez-Estrada M.T., McClements D.J., Decker E.A. (2009). Prooxidant mechanisms of free fatty acids in stripped soybean oil-in-water emulsions. J. Agric. Food Chem..

[8] Spitalniak-Bajerska K., Szumny A., Kucharska A.Z., Kupczyński R. (2018). Effect of natural antioxidants on the stability of linseed oil and fish stored under anaerobic conditions. J. Chem..

[9] Frankel E.N. (2012). Free radical oxidation. Lipid Oxidation.

[10] Rudzińska M., Przybylski R., Wąsowicz E. (2014). Degradation of phytosterols during storage of enriched margarines. Food Chem..

[11] Lanza B., Ninfali P. (2020). Antioxidants in extra virgin olive oil and table olives: Connections between agriculture and processing for health choices. Antioxidants.

[12] Oikawa S., Nishino K., Oikawa S., Inoue S., Mizutani T., Kawanishi S. (1998). Oxidative DNA damage and apoptosis induced by metabolites of butylated hydroxytoluene. Biochem. Pharmacol..

[13] Eskandani M., Hamishehkar H., Dolatabadi J.E.N. (2014). Cytotoxicity and DNA damage properties of tert-butylhydroquinone (TBHQ) food additive. Food Chem..

[14] Kawa-Rygielska J., Adamenko K., Kucharska A.Z., Szatkowska K. (2019). Fruit and herbal meads—Chemical composition and antioxidant properties. Food Chem..

[15] Bai Z., Yu R., Li J., Wang N., Wang Y., Niu L., Zhang Y. (2018). Application of several novel natural antioxidants to inhibit oxidation of tree peony seed oil. CyTA J. Food.

[16] Tura M., Mandrioli M., Gallina Toschi T. (2019). Preliminary study: Comparison of antioxidant activity of cannabidiol (CBD) and α-tocopherol added to refined olive and sunflower oils. Molecules.

[17] Cardenia V., Gallina Toschi T., Scappini S., Rubino R.C., Rodriguez-Estrada M.T. (2018). Development and validation of a fast gas chromatography/mass spectrometry method for the determination of cannabinoids in *Cannabis sativa* L.. J. Food Drug Anal..

[18] Pollastro F., Minassi A., Fresu L.G. (2018). Cannabis phenolics and their bioactivities. Curr. Med. Chem..

[19] Smeriglio A., Galati E.M., Monforte M.T., Lanuzza F., D’Angelo V., Circosta C. (2016). Polyphenolic compounds and antioxidant activity of cold-pressed seed oil from finola cultivar of *Cannabis sativa* L.. Phytother. Res..

[20] Lesma G., Consonni R., Gambaro V., Remuzzi C., Roda G., Silvani A., Vece V., Visconti G.L. (2014). Cannabinoid-free *Cannabis sativa* L. grown in the Po valley: Evaluation of fatty acid profile, antioxidant capacity and metabolic content. Nat. Prod. Res..

[21] Fiorini D., Molle A., Nabissi M., Santini G., Benelli G., Maggi F. (2019). Valorizing industrial hemp (*Cannabis sativa* L.) by-products: Cannabidiol enrichment in the inflorescence essential oil optimizing sample pre-treatment prior to distillation. Ind. Crops Prod..

[22] Leonard W., Zhang P., Ying D., Fang Z. (2020). Hempseed in food industry: Nutritional value, health benefits, and industrial applications. Compr. Rev. Food Sci. Food Saf..

[23] Izzo L., Castaldo L., Narváez A., Graziani G., Gaspari A., Rodríguez-Carrasco Y., Ritieni A. (2020). Analysis of phenolic compounds in commercial *Cannabis sativa* L. Inflorescences using UHPLC-Q-Orbitrap HRMS. Molecules.

[24] Bakro F., Jedryczka M., Wielgusz K., Sgorbini B., Inchingolo R., Cardenia V. (2020). Simultaneous determination of terpenes and cannabidiol in hemp (*Cannabis sativa* L.) by fast gas chromatography with flame ionization detection. J. Sep. Sci..

[25] Singleton V.L., Rossi J.A. (1965). Colorimetry of total phenolics with phosphomolybdic-phosphotungstic acid reagents. Am. J. Enol. Vitic..

[26] Barbosa-Pereira L., Guglielmetti A., Zeppa G. (2018). Pulsed electric field assisted extraction of bioactive compounds from cocoa bean shell and coffee silverskin. Food Bioprocess Technol..

[27] Yen G.C., Chung D.Y. (1999). Antioxidant effects of extracts from *Cassia tora* L. prepared under different degrees of roasting on the oxidative damage to biomolecules. J. Agric. Food Chem..

[28] Boon C.S., Xu Z., Yue X., McClements D.J., Weiss J., Decker E.A. (2008). Factors affecting lycopene oxidation in oil-in-water emulsions. J. Agric. Food Chem..

[29] Aksoz E., Korkut O., Aksit D., Gokbulut C. (2020). Vitamin E (α-, β + γ-and δ-tocopherol) levels in plant oils. Flavour Fragr. J..

[30] Toschi T.G., Cardenia V., Bonaga G., Mandrioli M., Rodriguez-Estrada M.T. (2014). Coffee silverskin: Characterization, possible uses, and safety aspects. J. Agric. Food Chem..

[31] European Commission (2002). Commission Regulation (EC) No 796/2002 of 6 May 2002 amending Regulation (EEC) No 2568/91 on the characteristics of olive oil and olive-pomace oil and on the relevant methods of analysis and the additional notes in the Annex to Council Regulation (EEC) No 2658/87 on the tariff and statistical nomenclature and on the Common Customs Tariff. Off. J. Eur. Union.

[32] Kamvissis V.N., Barbounis E.G., Megoulas N.C., Koupparis M.A. (2008). A Novel photometric method for evaluation of the oxidative stability of virgin olive oils. J. AOAC Int..

[33] Wang Y.Z., Fu S.G., Wang S.Y., Yang D.J., Wu Y.H.S., Chen Y.C. (2018). Effects of a natural antioxidant, polyphenol-rich rosemary (*Rosmarinus officinalis* L.) extract, on lipid stability of plant-derived omega-3 fatty-acid rich oil. LWT Food Sci. Technol..

[34] Barbosa-Pereira L., Rojo-Poveda O., Ferrocino I., Giordano M., Zeppa G. (2019). Assessment of volatile fingerprint by HS-SPME/GC-qMS and E-nose for the classification of cocoa bean shells using chemometrics. Food Res. Int..

[35] Drinić Z., Vidović S., Vladić J., Koren A., Kiprovski B., Sikora V. (2018). Effect of extraction solvent on total polyphenols content and antioxidant activity of *Cannabis sativa* L.. Lekovite Sirovine.

[36] (2017). Legge 2 dicembre 2016; n. 242. Disposizioni per la promozione della coltivazione e della filiera agroindustriale della canapa (16G00258) (GU n. 304 del 30/12/2016). Gazz. Uff. della Repubb. Ital..

[37] Hartsel J.A., Eades J., Hickory B., Makriyannis A. (2016). Cannabis sativa and hemp. Nutraceuticals.

[38] Andre C.M., Hausman J.-F., Guerriero G. (2016). *Cannabis sativa*: The plant of the thousand and one molecules. Front. Plant Sci..

[39] Nuutinen T. (2018). Medicinal properties of terpenes found in *Cannabis sativa* and *Humulus lupulus*. Eur. J. Med. Chem..

[40] Nissen L., Zatta A., Stefanini I., Grandi S., Sgorbati B., Biavati B., Monti A. (2010). Characterization and antimicrobial activity of essential oils of industrial hemp varieties (*Cannabis sativa* L.). Fitoterapia.

[41] Zhang K., Yao L. (2018). The anxiolytic effect of *Juniperus virginiana* L. essential oil and determination of its active constituents. Physiol. Behav..

[42] Cantele C., Rojo-Poveda O., Bertolino M., Ghirardello D., Cardenia V., Barbosa-Pereira L., Zeppa G. (2020). In vitro bioaccessibility and functional properties of phenolic compounds from enriched beverages based on cocoa bean shell. Foods.

[43] Frassinetti S., Moccia E., Caltavuturo L., Gabriele M., Longo V., Bellani L., Giorgi G., Giorgetti L. (2018). Nutraceutical potential of hemp (*Cannabis sativa* L.) seeds and sprouts. Food Chem..

[44] Manosroi A., Chankhampan C., Kietthanakorn B.O., Ruksiriwanich W., Chaikul P., Boonpisuttinant K., Sainakham M., Manosroi W., Tangjai T., Manosroi J. (2019). Pharmaceutical and cosmeceutical biological activities of hemp (*Cannabis sativa* L. var. *sativa*) leaf and seed extracts. Chiang Mai J. Sci..

[45] Chen T., He J., Zhang J., Li X., Zhang H., Hao J., Li L. (2012). The isolation and identification of two compounds with predominant radical scavenging activity in hempseed (seed of *Cannabis sativa* L.). Food Chem..

[46] Frankel E.N. (2012). Hydroperoxide decomposition. Lipid Oxidation.

[47] Malvis A., Šimon P., Dubaj T., Sládková A., Ház A., Jablonsky M., Sekretár S., Schmidt Š., Kreps F., Burčová Z. (2019). Determination of the thermal oxidation stability and the kinetic parameters of commercial extra virgin olive oils from different varieties. J. Chem..

[48] Davies C., Steele R. (2004). Lipolysis in lipid oxidation. Understanding and Measuring the Shelf-Life of Food.

[49] Mohanan A., Nickerson M.T., Ghosh S. (2018). Oxidative stability of flaxseed oil: Effect of hydrophilic, hydrophobic and intermediate polarity antioxidants. Food Chem..

[50] Martin-Rubio A.S., Sopelana P., Ibargoitia M.L., Guillén M.D. (2018). Prooxidant effect of α-tocopherol on soybean oil. Global monitoring of its oxidation process under accelerated storage conditions by 1H nuclear magnetic resonance. Food Chem..

[51] Buscato M.H.M., Müller F., Vetter W., Weiss J., Salminen H. (2020). Furan fatty acids in enriched ω-3 fish oil: Oxidation kinetics with and without added monomethyl furan fatty acid as potential natural antioxidant. Food Chem..

[52] Gordon M.H., Steele R. (2004). Factors affecting lipid oxidation. Understanding and Measuring the Shelf-Life of Food.

[53] Ma L., Liu G., Cheng W., Liu X., Brennan C., Brennan M.A., Liu H., Wang Q. (2020). The effect of heating on the formation of 4-hydroxy-2-hexenal and 4-hydroxy-2-nonenal in unsaturated vegetable oils: Evaluation of oxidation indicators. Food Chem..

[54] Huang S.W., Frankel E.N., German J.B. (1994). Antioxidant activity of α-and γ-tocopherols in bulk oils and in oil-in-water emulsions. J. Agric. Food Chem..

[55] Wojtunik K.A., Ciesla L.M., Waksmundzka-Hajnos M. (2014). Model studies on the antioxidant activity of common terpenoid constituents of essential oils by means of the 2,2-Diphenyl-1-picrylhydrazyl method. J. Agric. Food Chem..

[56] Torres-Martínez R., García-Rodríguez Y.M., Ríos-Chávez P., Saavedra-Molina A., López-Meza J.E., Ochoa-Zarzosa A., Garciglia R.S. (2018). Antioxidant activity of the essential oil and its major terpenes of *Satureja macrostema* (Moc. and Sessé ex Benth.) Briq. Pharmacogn. Mag..

[57] Misharina T.A., Samusenko A.L. (2008). Antioxidant properties of essential oils from lemon, grapefruit, coriander, clove, and their mixtures. Appl. Biochem. Microbiol..

[58] Dahham S., Tabana Y., Iqbal M., Ahamed M., Ezzat M., Majid A., Majid A. (2015). The anticancer, antioxidant and antimicrobial properties of the sesquiterpene β-caryophyllene from the essential oil of *Aquilaria crassna*. Molecules.

[59] Hacke A.C.M., Lima D., de Costa F., Deshmukh K., Li N., Chow A.M., Marques J.A., Pereira R.P., Kerman K. (2019). Probing the antioxidant activity of Δ9-tetrahydrocannabinol and cannabidiol in *Cannabis sativa* extracts. Analyst.

