# Supplementary material for: Antioxidant Effects of Hemp (Cannabis sativa L.) Inflorescence Extract in Stripped Linseed Oil
Source: Antioxidants (Basel). 2020 Nov 14;9(11):1131. doi: 10.3390/antiox9111131 (PMC7697792; doi:10.3390/antiox9111131)
Supplement: Supplementary file 1 [file antioxidants-09-01131-s001.pdf]

antioxidants

MDPI

Table S1. Terpenes profile of hemp inflorescences ethanolic extract. Values in mg/mL of extract are reported as mean ± standard deviation of three independent replicates (n=3).

Terpenoid	mg/mL of extract	% on the total
Monoterpenoids		
β-Pinene	1.69 ± 0.45	28.17
Limonene	0.41 ± 0.06	6.78
α-Pinene	0.30 ± 0.24	4.95
Linalool	0.23 ± 0.02	3.88
γ-Terpineol	0.15 ± 0.01	2.50
Borneol	0.06 ± 0.01	1.07
Fenchol	0.04 ± 0.02	0.63
Camphor	0.02 ± 0.00	0.37
Terpinolene	0.02 ± 0.00	0.32
Menthol	0.01 ± 0.00	0.10
Sum	2.92 ± 0.78	48.76
Sesquiterpenoids		
Cedrol	0.87 ± 0.07	14.49
β-Caryophyllene	0.72 ± 0.06	12.08
α-Bisabolol	0.48 ± 0.07	8.03
Eudesmol	0.33 ± 0.04	5.45
α-Humulene	0.26 ± 0.02	4.30
cis-Nerolidol	0.20 ± 0.02	3.30
Sum	2.86 ± 0.13	47.65
Diterpenoids		
Phytol	0.22 ± 0.03	3.59
Total	6.00 ± 0.73	

Table S2. Main lipid classes (g/100 g oil) in bulk oil without antioxidants (CO), with hemp extract (HO) and with α-tocopherol (EO). Values are reported as mean ± standard deviation of three independent replicates (n=3).

	Day	CO	HO	EO	Sig.
Free Fatty Acids (FFAs)	0	1.16 ± 0.03 ^{c}	1.23 ± 0.01	1.20 ± 0.02 ^{d}	n.s.
	0.25	1.17 ± 0.09 ^{bc}	1.16 ± 0.03	1.10 ± 0.09 ^{d}	n.s.
	1	1.19 ± 0.05 ^{bc}	1.23 ± 0.03	1.22 ± 0.03 ^{d}	n.s.
	2	1.18 ± 0.06 ^{bcY}	1.25 ± 0.05 ^{XY}	1.38 ± 0.01 ^{cX}	*
	3	1.43 ± 0.03 ^{aXY}	1.27 ± 0.01 ^{Y}	1.56 ± 0.08 ^{bX}	*
	5	1.32 ± 0.06 ^{abY}	1.21 ± 0.04 ^{Y}	1.80 ± 0.08 ^{aX}	**
	7	1.34 ± 0.08 ^{aY}	1.20 ± 0.01 ^{Y}	1.71 ± 0.11 ^{abX}	*
	Sig.	*	n.s.	***	
Free Sterols	0	0.32 ± 0.01	0.31 ± 0.01	0.33 ± 0.01	n.s.
	0.25	0.31 ± 0.03	0.33 ± 0.03	0.33 ± 0.02	n.s.
	1	0.33 ± 0.02	0.31 ± 0.01	0.33 ± 0.02	n.s.
	2	0.33 ± 0.00	0.33 ± 0.01	0.35 ± 0.02	n.s.
	3	0.33 ± 0.03	0.31 ± 0.01	0.36 ± 0.09	n.s.
	5	0.34 ± 0.01	0.32 ± 0.01	0.36 ± 0.04	n.s.
	7	0.34 ± 0.01	0.32 ± 0.00	0.34 ± 0.01	n.s.
	Sig.	n.s.	n.s.	n.s.	
Diacylglycerols (DAGs)	0	3.75 ± 0.05 ^{c}	3.92 ± 0.06	3.78 ± 0.21 ^{e}	n.s.
	0.25	3.79 ± 0.07 ^{c}	3.94 ± 0.19	3.87 ± 0.04 ^{de}	n.s.
	1	3.88 ± 0.26 ^{bc}	4.05 ± 0.01	4.27 ± 0.39 ^{cde}	n.s.
	2	4.18 ± 0.12 ^{abcXY}	3.85 ± 0.13 ^{Y}	4.51 ± 0.03 ^{cdX}	*
	3	4.51 ± 0.22 ^{aXY}	4.05 ± 0.11 ^{Y}	4.70 ± 0.18 ^{bcX}	*
	5	4.27 ± 0.08 ^{ab}	4.26 ± 0.16	5.22 ± 0.58 ^{ab}	n.s.
	7	4.50 ± 0.26 ^{aY}	4.25 ± 0.25 ^{Y}	5.40 ± 0.04 ^{aX}	*
	Sig.	*	n.s.	**	
Esterified Sterols	0	2.10 ± 0.22	2.02 ± 0.25	1.97 ± 0.17	n.s.
	0.25	2.09 ± 0.25	2.04 ± 0.58	2.15 ± 0.67	n.s.
	1	1.92 ± 0.23	1.71 ± 0.01	1.74 ± 0.01	n.s.
	2	2.25 ± 0.38	2.01 ± 0.54	2.05 ± 0.44	n.s.
	3	2.06 ± 0.25	1.81 ± 0.12	2.00 ± 0.37	n.s.
	5	2.33 ± 0.28	1.97 ± 0.44	2.02 ± 0.25	n.s.
	7	2.20 ± 0.22	2.03 ± 0.02	2.37 ± 0.30	n.s.
	Sig.	n.s.	n.s.	n.s.	
Triacylglycerols (TAGs)	0	92.67 ± 0.15 ^{a}	92.52 ± 0.31	92.72 ± 0.07 ^{a}	n.s.
	0.25	92.64 ± 0.06 ^{a}	92.52 ± 0.32	92.54 ± 0.24 ^{ab}	n.s.
	1	92.68 ± 0.05 ^{a}	92.70 ± 0.00	92.44 ± 0.43 ^{ab}	n.s.
	2	92.06 ± 0.20 ^{b}	92.56 ± 0.63	91.71 ± 0.39 ^{bc}	n.s.
	3	91.68 ± 0.04 ^{bY}	92.56 ± 0.01 ^{X}	91.38 ± 0.36 ^{cdY}	*
	5	91.75 ± 0.31 ^{bXY}	92.24 ± 0.33 ^{X}	90.60 ± 0.45 ^{deY}	*
	7	91.61 ± 0.12 ^{bX}	92.21 ± 0.29 ^{X}	90.18 ± 0.14 ^{eY}	**
	Sig.	**	n.s.	***	

Data were analyzed by one-way ANOVA. n.s. = not significant; * = $p < 0.05$; ** = $p < 0.01$; *** = $p < 0.001$.
Different lowercase letter in the same column denotes significant differences between the days of storage ($p < 0.05$; Duncan's test); different uppercase letter in the same row denotes significant differences between the bulk oils ($p < 0.05$; Duncan's test)

Table S3. Content of cannabidiol (CBD) (g/100 g oil) in bulk oil with hemp extract (HO). Values are reported as mean ± standard deviation of three independent replicates (n=3) and results of ANOVA analysis with Duncan's test (*p* < 0.05*)*

Day	CBD
0	0.40 ± 0.08
0.25	0.39 ± 0.01
1	0.41 ± 0.07
2	0.41 ± 0.06
3	0.40 ± 0.05
5	0.42 ± 0.10
7	0.41 ± 0.06
Sig.	n.s.

n.s. = not significant

Table S4. Total fatty acid methyl esters (FAMEs; g/100 g oil) in bulk oil without antioxidants (CO), during the storage treatment. Values are reported as mean ± standard deviation of three independent replicates (n=3).

FAME	Day 0	Day 0.25	Day 1	Day 2	Day 3	Day 5	Day 7	*Sig.*
C16:0	5.51 ± 0.01 [ab]	5.51 ± 0.02 [ab]	5.51 ± 0.02 [c]	5.52 ± 0.01 [ab]	5.64 ± 0.03 [a]	5.55 ± 0.02 [ab]	5.56 ± 0.02 [b]	**
C18:0	4.42 ± 0.01	4.44 ± 0.03	4.44 ± 0.01	4.43 ± 0.02	4.60 ± 0.08	4.46 ± 0.03	4.48 ± 0.03	n.s.
C20:0	0.13 ± 0.00	0.13 ± 0.00	0.13 ± 0.00	0.13 ± 0.00	0.14 ± 0.00	0.13 ± 0.00	0.13 ± 0.00	n.s.
C22:0	0.13 ± 0.00	0.13 ± 0.00	0.13 ± 0.00	0.13 ± 0.01	0.13 ± 0.00	0.13 ± 0.00	0.13 ± 0.01	n.s.
C18:1 *n*-9	16.98 ± 0.00 [b]	16.97 ± 0.07 [b]	17.01 ± 0.01 [b]	17.02 ± 0.04 [ab]	17.03 ± 0.02 [ab]	17.06 ± 0.03 [ab]	17.11 ± 0.04 [a]	*
C18:1 *n-7*	0.72 ± 0.01	0.73 ± 0.03	0.73 ± 0.00	0.73 ± 0.00	0.73 ± 0.01	0.73 ± 0.01	0.73 ± 0.01	n.s.
C20:1	0.11 ± 0.00	0.11 ± 0.00	0.11 ± 0.00	0.11 ± 0.00	0.11 ± 0.00	0.11 ± 0.00	0.11 ± 0.00	n.s.
C18:2 *n*-6	44.62 ± 0.00	44.54 ± 0.02	44.59 ± 0.04	44.58 ± 0.02	44.41 ± 0.05	44.53 ± 0.07	44.54 ± 0.05	n.s.
C18:3 *n*-3	26.70 ± 0.03 [a]	26.68 ± 0.07 [a]	26.65 ± 0.00 [ab]	26.63 ± 0.03 [ab]	26.46 ± 0.04 [c]	26.56 ± 0.07 [bc]	26.45 ± 0.06 [c]	**
C18:3γ	0.10 ± 0.00	0.11 ± 0.00	0.11 ± 0.00	0.10 ± 0.00	0.10 ± 0.00	0.11 ± 0.00	0.10 ± 0.00	n.s.
ΣCLA	0.01 ± 0.00	0.01 ± 0.00	0.01 ± 0.01	0.02 ± 0.01	0.03 ± 0.03	0.03 ± 0.02	0.02 ± 0.02	n.s.
ΣCLnA	0.03 ± 0.00	0.08 ± 0.06	0.05 ± 0.00	0.04 ± 0.01	0.05 ± 0.01	0.05 ± 0.02	0.05 ± 0.01	n.s.
Others	0.54 ± 0.01	0.57 ± 0.02	0.55 ± 0.01	0.55 ± 0.00	0.57 ± 0.01	0.55 ± 0.02	0.57 ± 0.01	n.s.

ΣCLA, conjugated linoleic acid isomers; ΣCLnA, conjugated linolenic acid isomers; n.s. = not significant; * = $p < 0.05$; ** = $p < 0.01$. Data were analyzed by one-way ANOVA. Different letter in the same row denotes significant differences between the days of storage ($p < 0.05$; Duncan's test)

Table S5. Total fatty acid methyl esters (FAMEs; g/100 g oil) in bulk oil with hemp extract antioxidants (HO), during the storage treatment. Values are reported as mean ± standard deviation of three independent replicates (n=3).

FAME	Day 0	Day 0.25	Day 1	Day 2	Day 3	Day 5	Day 7	*Sig.*
C16:0	5.50 ± 0.02	5.49 ± 0.04	5.52 ± 0.02	5.51 ± 0.02	5.48 ± 0.10	5.52 ± 0.01	5.52 ± 0.05	n.s.
C18:0	4.41 ± 0.01	4.40 ± 0.04	4.43 ± 0.02	4.42 ± 0.02	4.41 ± 0.11	4.43 ± 0.02	4.43 ± 0.05	n.s.
C20:0	0.13 ± 0.00	0.13 ± 0.00	0.13 ± 0.00	0.27 ± 0.20	0.13 ± 0.00	0.13 ± 0.00	0.13 ± 0.00	n.s.
C22:0	0.10 ± 0.00	0.11 ± 0.00	0.11 ± 0.00	0.10 ± 0.00	0.11 ± 0.00	0.11 ± 0.01	0.10 ± 0.02	n.s.
C18:1 *n*-9	16.96 ± 0.00	16.93 ± 0.07	16.99 ± 0.01	16.96 ± 0.04	17.02 ± 0.01	16.98 ± 0.01	17.00 ± 0.04	n.s.
C18:1 *n*-7	0.73 ± 0.01	0.73 ± 0.01	0.73 ± 0.00	0.73 ± 0.00	0.73 ± 000	0.73 ± 0.01	0.74 ± 0.00	n.s.
C20:1	0.11 ± 0.00	0.11 ± 0.00	0.11 ± 0.00	0.11 ± 0.00	0.11 ± 0.00	0.11 ± 0.00	0.11 ± 0.00	n.s.
C18:2 *n*-6	44.59 ± 0.01	44.59 ± 0.04	44.58 ± 0.04	44.39 ± 0.06	44.59 ± 0.16	44.53 ± 0.04	44.50 ± 0.02	n.s.
C18:3 *n*-3	26.71 ± 0.00	26.78 ± 0.12	26.67 ± 0.01	26.56 ± 0.01	26.67 ± 0.07	26.68 ± 0.01	26.64 ± 0.04	n.s.
C18:3γ	0.10 ± 0.00	0.11 ± 0.00	0.11 ± 0.00	0.10 ± 0.00	0.11 ± 0.00	0.11 ± 0.00	0.10 ± 0.00	n.s.
ΣCLA	0.02 ± 0.01	0.01 ± 0.01	0.02 ± 0.01	0.02 ± 0.01	0.02 ± 0.01	0.03 ± 0.00	0.06 ± 0.05	n.s.
ΣCLnA	0.05 ± 0.00	0.04 ± 0.02	0.04 ± 0.00	0.21 ± 0.24	0.05 ± 0.01	0.05 ± 0.01	0.06 ± 0.03	n.s.
Others	0.56 ± 0.00	0.55 ± 0.01	0.55 ± 0.00	0.58 ± 0.02	0.56 ± 0.00	0.56 ± 0.01	0.55 ± 0.01	n.s.

ΣCLA, sum of conjugated linoleic acid isomers; ΣCLnA, sum of conjugated linolenic acid isomers; n.s. = not significant; data were analyzed by one-way ANOVA ($p < 0.05$; Duncan's test)

Table S6. Total fatty acid methyl esters (FAMEs; g/100 g oil) in bulk oil with α-tocopherol (EO) during the storage treatment. Values are reported as mean ± standard deviation of three independent replicates (n=3).

FAME	Day 0	Day 0.25	Day 1	Day 2	Day 3	Day 5	Day 7	*Sig.*
C16:0	5.49 ± 0.00 d	5.51 ± 0.02 de	5.53 ± 0.02 de	5.55 ± 0.01 cd	5.57 ± 0.02 bc	5.62 ± 0.02 a	5.61 ± 0.03 ab	**
C18:0	4.41 ± 0.00 d	4.43 ± 0.01 cd	4.44 ± 0.01 bcd	4.46 ± 0.01 bc	4.48 ± 0.03 ab	4.53 ± 0.03 a	4.53 ± 0.03 a	**
C20:0	0.13 ± 0.00 b	0.13 ± 0.00 ab	0.13 ± 0.00 ab	0.13 ± 0.00 ab	0.13 ± 0.00 ab	0.13 ± 0.00 ab	0.14 ± 0.00 a	*
C22:0	0.13 ± 0.00 b	0.13 ± 0.00 b	0.13 ± 0.00 b	0.13 ± 0.00 ab	0.13 ± 0.00 ab	0.13 ± 0.00 ab	0.15 ± 0.02 a	n.s.
C18:1 *n*-9	16.99 ± 0.00 e	17.01 ± 0.00 de	17.05 ± 0.00 d	17.11 ± 0.01 c	17.19 ± 0.02 b	17.32 ± 0.05 a	17.30 ± 0.03 a	***
C18:1 *n*-7	0.73 ± 0.00	0.72 ± 0.00	0.73 ± 0.00	0.74 ±0.01	0.75 ± 0.00	0.74 ± 0.01	0.74 ± 0.01	n.s.
C20:1	0.11 ± 0.00 a	0.11 ± 0.00 ab	0.11 ± 0.00 ab	0.11 ± 0.00 ab	0.11 ± 0.00 ab	0.12 ± 0.00 ab	0.12 ± 0.00 b	n.s.
C18:2 *n*-6	44.63 ± 0.02 a	44.61 ± 0.04 ab	44.59 ± 0.04 ab	44.59 ± 0.04 ab	44.57 ± 0.01 ab	44.60 ± 0.04 ab	44.54 ± 0.02 b	*
C18:3 *n*-3	26.68 ± 0.00 a	26.64 ± 0.02 a	26.56 ± 0.01 b	26.45 ± 0.02 c	26.29 ± 0.01 d	26.08 ± 0.06 e	26.12 ± 0.01 e	***
C18:3*γ*	0.10 ± 0.00	0.11 ± 0.00	0.11 ± 0.00	0.10 ± 0.00	0.10 ± 0.00	0.10 ± 0.00	0.10 ± 0.00	n.s.
ΣCLA	0.01 ± 0.00	0.01 ± 0.00	0.01 ± 0.01	0.02 ± 0.00	0.01 ± 0.01	0.01 ± 0.00	0.04 ± 0.03	n.s.
ΣCLnA	0.04 ± 0.02	0.05 ± 0.03	0.05 ± 0.00	0.04 ± 0.00	0.07 ± 0.06	0.05 ± 0.02	0.05 ± 0.02	n.s.
Others	0.56 ± 0.00	0.55 ± 0.01	0.56 ± 0.01	0.56 ± 0.01	0.57 ± 0.00	0.57 ± 0.01	0.57 ± 0.00	n.s.

ΣCLA, sum of conjugated linoleic acid isomers; ΣCLnA, sum of conjugated linolenic acid isomers; n.s. = not significant; * = $p < 0.05$; ** = $p < 0.01$; *** = $p < 0.001$. Data were analyzed by one-way ANOVA. Different letter in the same row denotes significant differences between the days of storage ($p < 0.05$; Duncan's test)

Table S7. Peroxide value (meq O_2/kg oil) in bulk oil without antioxidants (CO), with hemp extract (HO) and with α-tocopherol (EO). Values are reported as mean ± standard deviation of three independent replicates (n=3).

Day	CO	HO	EO	*Sig.*
0	2.45 ± 0.02 ^{d}	3.26 ± 0.18 ^{d}	3.91 ± 0.66 ^{d}	n.s.
0.25	3.14 ± 0.11 ^{dX}	2.92 ± 0.06 ^{dX}	7.63 ± 0.52 ^{cY}	***
1	6.71 ± 0.22 ^{dX}	8.24 ± 1.77 ^{cdX}	25.22 ± 0.94 ^{bY}	***
2	17.83 ± 0.95 ^{cX}	13.65 ± 2.20 ^{bcX}	45.46 ± 0.74 ^{aY}	***
3	30.95 ± 8.03 ^{b}	17.57 ± 3.17 ^{ab}	n.a.	n.s.
5	37.52 ± 2.62 ^{b}	16.02 ± 2.97 ^{ab}	n.a.	*
7	49.57 ± 0.22 ^{a}	21.19 ± 1.44 ^{a}	n.a.	***
Sig.	***	***	***	

Data were analyzed by one-way ANOVA. n.s. = not significant; * = $p < 0.05$; *** = $p < 0.001$.
Different lowercase letter in the same column denotes significant differences between the days of storage ($p < 0.05$; Duncan's test); different uppercase letter in the same row denotes significant differences between the bulk oils ($p < 0.05$; Duncan's test)

Table S8. Formation of hexanal (mmol/kg oil) in bulk oil without antioxidants (CO), with hemp extract (HO) and with α-tocopherol (EO). Values are reported as mean ± standard deviation of three independent replicates (n=3).

Day	CO	HO	EO	*Sig.*
0	8.71 ± 0.13 ^{cd}	6.43 ± 2.71	3.57 ± 0.66 ^{d}	n.s.
0.25	7.93 ± 0.34 ^{d}	7.21 ± 0.81	4.48 ± 0.52 ^{d}	n.s.
1	11.77 ± 0.02 ^{cdX}	3.34 ± 0.52 ^{Y}	5.34 ± 0.94 ^{dY}	**
2	15.20 ± 0.58 ^{cX}	5.72 ± 1.52 ^{Y}	5.29 ± 0.74 ^{dY}	**
3	41.88 ± 2.76 ^{bX}	6.32 ± 0.46 ^{Y}	8.45 ± 1.01 ^{cY}	***
5	42.17 ± 3.04 ^{bX}	3.15 ± 0.11 ^{Z}	13.28 ± 0.82 ^{bY}	***
7	69.02 ± 5.98 ^{aX}	7.67 ± 0.95 ^{Z}	26.53 ± 0.54 ^{aY}	***
Sig.	***	n.s.	***	

Data were analyzed by one-way ANOVA. n.s. = not significant; ** = $p < 0.01$; *** = $p < 0.001$.

Different lowercase letter in the same column denotes significant differences between the days of storage ($p < 0.05$; Duncan's test); different uppercase letter in the same row denotes significant differences between the bulk oils ($p < 0.05$; Duncan's test)

Table S9. Identified volatile compounds (as peak area amount) in thermoxidized stripped oil samples without hemp extract or α-tocopherol (CO) for 0, 0.25, 1, 2, 3, 5 and 7 days.

Volatile compounds	Day 0	Day 0.25	Day 1	Day 2	Day 3	Day 5	Day 7
Decane	400869 ± 58740	410047 ± 3354	445648 ± 53702	566379 ± 116280	651843 ± 29739	79214 ± 6019	683713 ± 210444
α-Pinene	nd	nd	nd	nd	nd	nd	nd
2-Butenal	32702 ± 9129	48816 ± 3844	150348 ± 139743	639210 ± 105457	766814 ± 150897	1042673 ± 273808	3229981 ± 1038232
Hexanal	333841 ± 83488	139904 ± 20848	589491 ± 32656	711505 ± 105123	1933731 ± 165095	866977 ± 212103	2901713 ± 652714
Undecane	227830 ± 68259	239583 ± 62069	285235 ± 19203	332507 ± 18168	378527 ± 50361	291806 ± 11704	373007 ± 76911
β-Pinene	nd	nd	nd	nd	nd	nd	nd
p-Xylene	nd	nd	nd	nd	nd	nd	nd
3-Carene	nd	nd	nd	nd	nd	nd	nd
β-Myrcene	nd	nd	nd	nd	nd	nd	nd
Limonene	nd	nd	nd	nd	nd	nd	nd
o-Cymene	nd	nd	nd	nd	nd	nd	nd
2,4-Nonadiene	5477 ± 1597	8219 ± 1315	13813 ± 1236	31434 ± 5230	61782 ± 26220	87594 ± 20660	314009 ± 137858
trans-3-Caren-2-ol	nd	nd	nd	nd	nd	nd	nd
4-Carene	nd	nd	nd	nd	nd	nd	nd
Hexadecane	80097 ± 44910	148725 ± 6778	118930 ± 26777	25655 ± 5721	64871 ± 27255	58484 ± 39342	26502 ± 498
2,4-Diethyl-1-heptanol	23770 ± 1319	33390 ± 3381	34804 ± 9131	28018 ± 5083	33383 ± 8270	33777 ± 7478	22929 ± 754
6-Methyl-5-heptene	nd	nd	nd	nd	nd	nd	nd
1-Hexanol	1041476 ± 113776	1060787 ± 40497	1189612 ± 48266	1351131 ± 133259	1529637 ± 117991	1347319 ± 250761	1312877 ± 428929
4-Hydroxy-4-methyl-2-pentanone	148178 ± 131356	117772 ± 71046	295933 ± 217530	419647 ± 274190	92975 ± 43092	201889 ± 214631	125350 ± 54888
Nonanal	50390 ± 11162	296020 ± 104774	133199 ± 17417	172258 ± 59927	47727 ± 12034	72562 ± 14394	36079 ± 793
Fenchone	nd	nd	nd	nd	nd	nd	nd
Butanoic acid, hexyl ester	nd	nd	nd	nd	nd	nd	nd

1,3-Di-tert-butylbenzene	235373 ± 49979	263899 ± 50326	301203 ± 10658	286335 ± 32803	258242 ± 51697	254983 ± 19740	234302 ± 4243
trans-Linalool oxide	nd	nd	nd	nd	nd	nd	nd
Acetic acid	139423 ± 34288	145521 ± 27142	180310 ± 76342	430721 ± 16594	531045 ± 41269	566291 ± 117680	1646080 ± 53594
2,4-Heptadienal	24206 ± 12357	43645 ± 1949	75157 ± 46513	233865 ± 62225	332770 ± 156845	370722 ± 103052	1160636 ± 619028
Formic acid	45757 ± 59357	33086 ± 35746	90401 ± 116803	1157315 ± 288803	1308826 ± 609514	1722387 ± 1369303	7695097 ± 2721224
β-Linalool	nd	nd	nd	nd	nd	nd	nd
trans-2-Pinanol	nd	nd	nd	nd	nd	nd	nd
2-Norbornanol	nd	nd	nd	nd	nd	nd	nd
Camphene hydrate	nd	nd	nd	nd	nd	nd	nd
Caryophyllene	nd	nd	nd	nd	nd	nd	nd
β-Farnesene	nd	nd	nd	nd	nd	nd	nd
Ipsedienol	nd	nd	nd	nd	nd	nd	nd
α-Humulene	nd	nd	nd	nd	nd	nd	nd
α-Terpineol	nd	nd	nd	nd	nd	nd	nd
Borneol	188347 ± 23041	234595 ± 40961	258567 ± 10269	257979 ± 30498	244629 ± 42190	223214 ± 24329	217915 ± 10459
Guaiol	nd	nd	nd	nd	nd	nd	nd

The results are expressed as mean ± standard deviation. Abbreviations: nd, not detected

Table S10. Identified volatile compounds (as peak area amount) in thermoxidized stripped oil samples containing hemp extract (HO) for 0, 0.25, 1, 2, 3, 5 and 7 days.

Volatile compounds	Day 0	Day 0.25	Day 1	Day 2	Day 3	Day 5	Day 7
Decane	nd	nd	nd	nd	nd	nd	nd
α-Pinene	471329 ± 48905	523860 ± 3941	584049 ± 16983	570395 ± 15957	567174 ± 55016	698279 ± 167422	656200 ± 239173
2-Butenal	nd	nd	nd	nd	nd	nd	nd
Hexanal	139991 ± 8698	506292 ± 280274	175174 ± 20476	388634 ± 207229	353925 ± 246322	145191 ± 50883	279995 ± 130296
Undecane	169104 ± 89198	182212 ± 44335	258377 ± 29694	285737 ± 23026	306225 ± 66111	506952 ± 427063	281789 ± 969
β-Pinene	1428932 ± 606208	1465797 ± 513654	1554376 ± 442639	1837414 ± 1039875	1832421 ± 440010	1763933 ± 156693	1525635 ± 717534

p-Xylene	133178 ± 5154	131944 ± 19462	148306 ± 33596	165702 ± 26787	169493 ± 30506	158903 ± 65923	144139 ± 9499
3-Carene	57695 ± 34	62602 ± 14066	76952 ± 10583	77637 ± 15481	77752 ± 13186	57622 ± 4748	67268 ± 5372
β-Myrcene	15520832 ± 5382425	15576341 ± 4380295	17171609 ± 3517797	18452524 ± 9024216	19789881 ± 3588207	17612008 ± 798436	17611918 ± 6629021
Limonene	5681981 ± 1084186	5753071 ± 854382	6187013 ± 375838	6776789 ± 2258475	7182218 ± 155093	6720827 ± 870052	6219773 ± 1441535
o-Cymene	153353 ± 17977	185349 ± 2526	156285 ± 16632	191015 ± 63817	198233 ± 9554	187009 ± 50582	167277 ± 23125
2,4-Nonadiene	8198 ± 2010	6392 ± 975	8645 ± 950	13494 ± 754	17943 ± 2246	21130 ± 3065	25619 ± 2203
trans-3-Caren-2-ol	247884 ± 5611	227695 ± 14356	220754 ± 25451	233282 ± 63702	282942 ± 59334	248745 ± 99044	221231 ± 25554
4-Carene	91504 ± 10063	98097 ± 5532	90637 ± 6437	90723 ± 14122	102505 ± 22770	86822 ± 22193	92698 ± 7570
Hexadecane	241786 ± 17872	375160 ± 192808	274925 ± 28201	281794 ± 11565	334329 ± 17900	329250 ± 91207	309451 ± 43860
2,4-Diethyl-1-heptanol	36709 ± 8880	34200 ± 3678	43937 ± 7984	39426 ± 6424	46400 ± 16118	32976 ± 6251	55100 ± 4214
6-Methyl-5-heptene	21206 ± 1860	21642 ± 392	21806 ± 6418	44428 ± 840	58053 ± 20613	34263 ± 3225	43214 ± 8331
1-Hexanol	801031 ± 49965	774719 ± 102506	849659 ± 168532	961544 ± 234061	1114824 ± 329376	1245213 ± 868081	802940 ± 84064
4-Hydroxy-4-methyl-2-pentanone	126656 ± 53124	126238 ± 17052	164099 ± 95558	53633 ± 28290	378312 ± 179686	197097 ± 7450	104532 ± 9643
Nonanal	65687 ± 42269	414744 ± 525384	35183 ± 3869	63914 ± 2179	39710 ± 13054	177767 ± 203221	30855 ± 744
Fenchone	751715 ± 18664	731240 ± 90796	789556 ± 90148	846515 ± 155996	908933 ± 145090	812938 ± 229275	704212 ± 73833
Butanoic acid, hexyl ester	152572 ± 34066	172982 ± 26397	177968 ± 13074	196988 ± 51598	219829 ± 9328	203456 ± 36659	185963 ± 44879
1,3-Di-tert-butylbenzene	292090 ± 18718	302992 ± 62525	301849 ± 67309	297012 ± 19481	320757 ± 87430	265866 ± 41750	274376 ± 30187
trans-Linalool oxide	89805 ± 8788	99437 ± 2620	100929 ± 12455	109681 ± 24798	114305 ± 49	117036 ± 38817	102208 ± 15738
Acetic acid	679569 ± 41775	831220 ± 213070	857452 ± 193861	898543 ± 491795	1204286 ± 249297	1640522 ± 1189020	1028847 ± 61812
2,4-Heptadienal	nd	nd	nd	nd	nd	nd	nd
Formic acid	nd	nd	nd	nd	nd	nd	nd
β-Linalool	2272809 ± 115827	2385387 ± 79810	2535152 ± 292733	2664161 ± 228042	2912362 ± 345098	2515135 ± 263043	2653388 ± 148073
trans-2-Pinanol	643125 ± 24662	637583 ± 51055	708405 ± 102827	748355 ± 119411	769606 ± 47689	713536 ± 147677	710230 ± 47401
2-Norbornanol	2381412 ± 212381	2552651 ± 202508	2658099 ± 343387	2769975 ± 321255	3039547 ± 520887	2645576 ± 586597	2501479 ± 107417
Camphene hydrate	347947 ± 27444	286781 ± 39616	273285 ± 48472	255451 ± 26441	238011 ± 19060	183596 ± 29586	229897 ± 10139

Caryophyllene	863454	±	66712	989021	±	132136	1030017	±	204234	1022876	±	151329	1061669	±	263957	941628	±	157637	971466	±	165025
β-Farnesene	65174	±	17027	70817	±	24521	62679	±	8409	74254	±	10723	81277	±	16865	87579	±	34329	80005	±	3895
Ipsedienol	449810	±	7369	423767	±	13462	474985	±	5561	506156	±	171309	544435	±	56025	470298	±	15468	514734	±	117169
α-Humulene	744120	±	54036	820816	±	192776	797439	±	177936	790640	±	133519	809625	±	215839	746302	±	167594	744358	±	143427
α-Terpineol	1412775	±	53811	1496609	±	224831	1469090	±	190357	1477289	±	17203	1616130	±	217275	1425517	±	187377	1445160	±	5262
Borneol	1764518	±	68204	1839221	±	210396	1855251	±	229227	1870298	±	107265	2062076	±	326572	1775105	±	311795	1766932	±	35816
Guaiol	152106	±	15090	156072	±	18977	164521	±	13666	207080	±	32816	135367	±	1882	205335	±	94544	204833	±	5069

The results are expressed as mean ± standard deviation. Abbreviations: nd, not detecte

Table S11. Identified volatile compounds (as peak area amount) in thermoxidized stripped oil samples containing α-tocopherol (EO) for 0, 0.25, 1, 2, 3, 5 and 7 days.

Volatile compounds	Day 0			Day 0.25			Day 1			Day 2			Day 3			Day 5			Day 7		
Decane	402578	±	40084	443885	±	42846	512933	±	11533	518501	±	18516	534807	±	28762	600588	±	85331	626827	±	154075
α-Pinene		nd			nd			nd			nd			nd			nd			nd	
2-Butenal	10720	±	2681	32759	±	12002	78150	±	31899	129250	±	17043	379469	±	36939	305620	±	179099	591705	±	131818
Hexanal	139801	±	21039	164878	±	4045	273278	±	75072	246443	±	12617	408634	±	36869	667174	±	81039	1246142	±	46511
Undecane	227982	±	42827	231629	±	7529	253688	±	2798	259594	±	21792	282664	±	39785	305831	±	48710	309996	±	33869
β-Pinene		nd			nd			nd			nd			nd			nd			nd	
p-Xylene		nd			nd			nd			nd			nd			nd			nd	
3-Carene		nd			nd			nd			nd			nd			nd			nd	
β-Myrcene		nd			nd			nd			nd			nd			nd			nd	
Limonene		nd			nd			nd			nd			nd			nd			nd	
o-Cymene		nd			nd			nd			nd			nd			nd			nd	
2,4-Nonadiene	5898	±	1137	7303	±	744	13446	±	358	35080	±	169	100643	±	8602	141727	±	10399	305540	±	27910
trans-3-Caren-2-ol		nd			nd			nd			nd			nd			nd			nd	
4-Carene		nd			nd			nd			nd			nd			nd			nd	
Hexadecane	66143	±	22176	86700	±	16005	89956	±	5657	76014	±	12798	61611	±	16016	82108	±	2796	82970	±	208

2,4-Diethyl-1-heptanol	36815	±	9727	24617	±	1515	29191	±	1049	28358	±	4887	39752	±	23815	34978	±	18311	33930	±	3231
6-Methyl-5-heptene		nd			nd			nd			nd			nd			nd			nd	
1-Hexanol	992735	±	37667	1055633	±	40351	1171417	±	3143	1143731	±	46296	1145352	±	118717	1337341	±	275874	1252044	±	300679
4-Hydroxy-4-methyl-2-pentanone	23491	±	1549	40156	±	38568	27834	±	12948	34713	±	7070	58370	±	17127	40628	±	3262	75133	±	3777
Nonanal	37877	±	357	61373	±	11997	32883	±	6937	16312	±	1667	96968	±	1832	70329	±	3256	34324	±	4183
Fenchone		nd			nd			nd			nd			nd			nd			nd	
Butanoic acid, hexyl ester		nd			nd			nd			nd			nd			nd			nd	
1,3-Di-tert-butylbenzene	246349	±	55858	272760	±	1207	284039	±	44872	298524	±	11536	293095	±	62937	292352	±	87704	315850	±	53575
trans-Linalool oxide		nd			nd			nd			nd			nd			nd			nd	
Acetic acid	75652	±	17287	125131	±	6377	207757	±	8362	401334	±	25150	638124	±	89232	830888	±	52864	1287401	±	272894
2,4-Heptadienal	34524	±	7649	93110	±	3729	264413	±	69766	361121	±	7595	885534	±	41062	1082233	±	1061	1728166	±	128432
Formic acid	1785	±	2048	8168	±	943	88657	±	37085	505838	±	333310	1362264	±	94298	1794231	±	814796	5338211	±	2173005
β-Linalool		nd			nd			nd			nd			nd			nd			nd	
trans-2-Pinanol		nd			nd			nd			nd			nd			nd			nd	
2-Norbornanol		nd			nd			nd			nd			nd			nd			nd	
Camphene hydrate		nd			nd			nd			nd			nd			nd			nd	
Caryophyllene		nd			nd			nd			nd			nd			nd			nd	
β-Farnesene		nd			nd			nd			nd			nd			nd			nd	
Ipsedienol		nd			nd			nd			nd			nd			nd			nd	
α-Humulene		nd			nd			nd			nd			nd			nd			nd	
α-Terpineol		nd			nd			nd			nd			nd			nd			nd	
Borneol	99233	±	50311	124663	±	43712	166020	±	30724	213325	±	34802	201079	±	1014	241907	±	43897	252637	±	13748
Guaiol		nd			nd			nd			nd			nd			nd			nd	

The results are expressed as mean ± standard deviation. Abbreviations: nd, not detected.

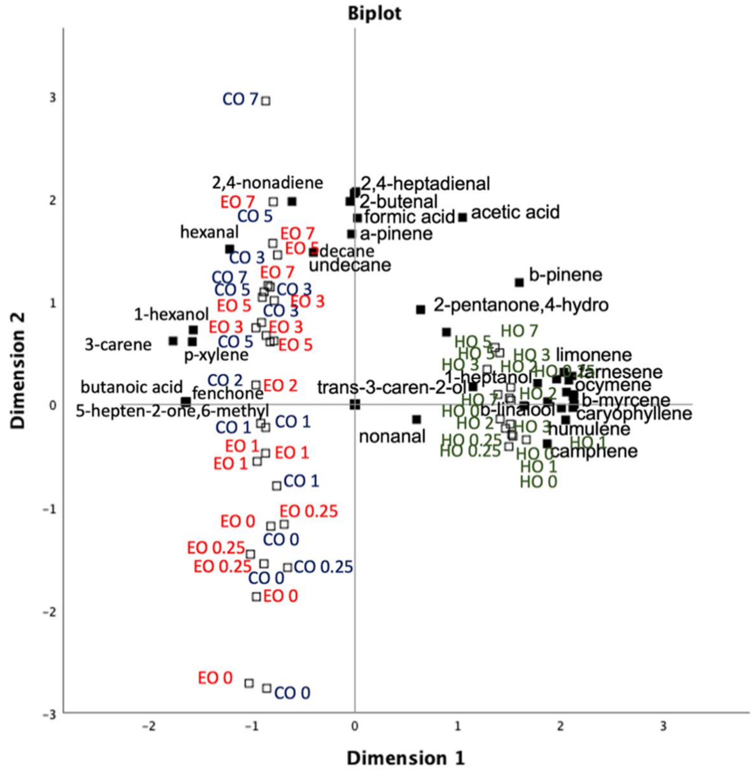

Figure S1. Bi-plot of all volatile compounds (black squares) and treatment time (empty squares) in thermo-oxidized bulk oils.
